# The proline-rich region of 18.5 kDa myelin basic protein binds to the SH3-domain of Fyn tyrosine kinase with the aid of an upstream segment to form a dynamic complex *in vitro*

**DOI:** 10.1042/BSR20140149

**Published:** 2014-12-08

**Authors:** Miguel De Avila, Kenrick A. Vassall, Graham S. T. Smith, Vladimir V. Bamm, George Harauz

**Affiliations:** *Department of Molecular and Cellular Biology, University of Guelph, 50 Stone Road East, Guelph, Ontario N1G 2W1, Canada

**Keywords:** amphipathic α-helix, intrinsically-disordered proteins, isothermal titration calorimetry (ITC), myelin basic protein, NMR spectroscopy, oligodendrocyte, poly-proline type II (PPII), SH3-domain, CARA, Computer Assisted Resonance Assignment, CNS, central nervous system, DAPI, 4′,6-diamidino-2-phenylindole, IDP, intrinsically disordered protein, ITC, isothermal titration calorimetry, MAP, mitogen-activated protein, MAPK, mitogen-activated protein kinase, MBP, myelin basic protein, NOE, nuclear Overhauser effect, PPII, poly-proline type II, RFP, red fluorescent protein, SH3, Src homology 3, SUMO, small ubiquitin-related modifier, ZO-1, zonula occludens 1

## Abstract

The intrinsically disordered 18.5 kDa classic isoform of MBP (myelin basic protein) interacts with Fyn kinase during oligodendrocyte development and myelination. It does so primarily via a central proline-rich SH3 (Src homology 3) ligand (T92–R104, murine 18.5 kDa MBP sequence numbering) that is part of a molecular switch due to its high degree of conservation and modification by MAP (mitogen-activated protein) and other kinases, especially at residues T92 and T95. Here, we show using co-transfection experiments of an early developmental oligodendroglial cell line (N19) that an MBP segment upstream of the primary ligand is involved in MBP–Fyn–SH3 association *in cellula*. Using solution NMR spectroscopy *in vitro*, we define this segment to comprise MBP residues (T62–L68), and demonstrate further that residues (V83–P93) are the predominant SH3-target, assessed by the degree of chemical shift change upon titration. We show by chemical shift index analysis that there is no formation of local poly-proline type II structure in the proline-rich segment upon binding, and by NOE (nuclear Overhauser effect) and relaxation measurements that MBP remains dynamic even while complexed with Fyn–SH3. The association is a new example first of a non-canonical SH3-domain interaction and second of a fuzzy MBP complex.

## Abstract 2

MBP interacts with Fyn kinase during oligodendrocyte development and myelination. We show that there is no binding-induced PPII formation in the primary ligand segment, and that a region upstream is required for in vitro interaction.

## INTRODUCTION

In the CNS (central nervous system), myelin is generated by oligodendrocytes and forms a multilamellar, periodic, lipid-rich structure that surrounds axons to facilitate saltatory conduction [1,2]. The adhesion of the cytoplasmic leaflets of compact myelin in the mature CNS is primarily carried out by MBP (myelin basic protein), which is a highly positively-charged, developmentally regulated protein family expressed from the gene in the oligodendrocyte lineage (*Golli*) [3,4]. Specifically, the 18.5 kDa size isoform has been deemed the ‘classic’ or ‘executive’ isoform since it is the most abundant in the adult human brain, and is essential for proper myelin membrane compaction [5]. As previously reviewed [4,6,7], the protein has many other binding partners: cytoskeletal proteins (actin, tubulin), Ca^2+^-activated calmodulin, and proteins containing SH3 (Src homology 3)-domains. The latter include mainly Fyn kinase [8–11], but also ZO-1 (zonula occludens 1) and cortactin [12,13]. This protein's multifunctionality in myelin is derived partly from its conformational plasticity as an IDP (intrinsically disordered protein) [6,7,14,15].

The interactions of IDPs with target proteins are complex, often involving interplays of disorder-to-order transitions that could represent either binding of transient secondary structure recognition motifs, or alternatively induced folding of these motifs upon binding, as recently reviewed and discussed critically [16,17]. The resultant complexes have been described as ‘fuzzy’, meaning that they are highly polymorphic [18,19]. Many binding motifs ‘moonlight’, i.e., have more than one potential interaction partner [20]. The classic isoforms of MBP are quintessential IDPs exhibiting all of these facets [4,6,7,14,15]. The interactions of 18.5 kDa MBP with membranes, actin, and Ca^2+^-calmodulin have been investigated in detail using diverse spectroscopies, and all involve some degree of local disorder-to-order transition (usually to an α-helix), yet with many segments remaining mobile and dynamic, i.e., ‘fuzzy’ [15,21].

We have also sought to characterize the interaction of MBP with SH3-domains using *in silico*, *in vivo* and *in vitro* experiments [8–12,22,23]. The SH3-domains are a ubiquitous protein-recognition module, most of which identify and bind proline-rich regions that can adopt a PPII (poly-proline type II) conformation, and a variety of target sequences, but often based on a P–x–x–P consensus [24–34]. Specificity in these interactions arises from slight variations in the consensus sequences of each SH3-domain, and the hydrophobic and charge interactions of the residues near the binding regions of each protein. These interactions usually have a *K*_D_ value in the low-micromolar range and follow a two-state model of binding [35]. Usually, a full characterization of the interactions between these domains and their binding partners is achievable *in vitro* by a combination of biochemical and biophysical methods used on the SH3-domain alone, and 7- to 30-residue peptides comprising the proline-rich region of the binding protein (e.g., [34–38]).

**Figure 1:**
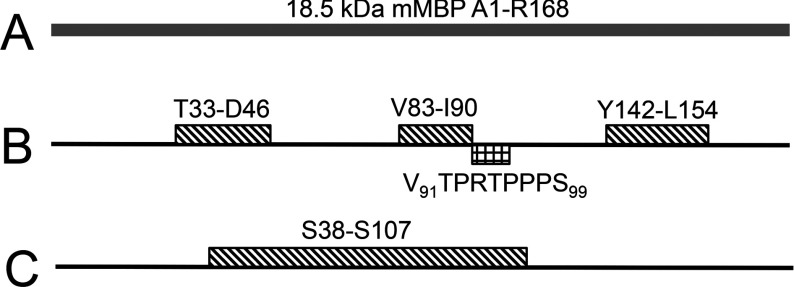
The α-helical and poly-proline type II regions of 18.5-kDa MBP, and peptide constructed for this study (**A**) Cartoon representation of the entire 18.5 kDa murine MBP sequence (168 residues). The full amino acid sequence is provided in Figure S1. (**B**) The three amphipathic α-helical regions, identified in MBP upon interaction with myelin-mimetic membranes by a variety of experimental techniques (represented by the rectangles with the diagonal-fill pattern). The box with the rectangular-fill pattern shows the location and sequence of the proline-rich region adjacent to the central α-helical region. (**C**) Representation of the *xα*2-peptide (residues (S38–S107) that was constructed here, in comparison to the known ordered secondary structural elements. This peptide has a molecular mass of 7642.4 Da and pI of 11.34, with net charge +8 at neutral pH (as calculated by the ProtParam module in www.expasy.ch).

The 18.5-kDa MBP isoform contains a proline-rich region (T92–P93–R94–T95–P96–P97–P98, murine numbering) immediately adjacent to a membrane-associated amphipathic α-helix (Figure 1, and Supplementary Figure S1). Both regions are part of a highly conserved central segment and likely comprise a molecular switch due to the presence of MAPK (mitogen-activated protein kinase) sites (T92 and T95), and the ability to undergo disorder-to-order transitions by adopting α-helical structure (reviewed in [4,7,14,15,21]). We have shown in previous studies involving the full-length protein as well as short MBP peptides (T92–R104, F86–G103, E80–G103 and S72–S107) that the proline-rich region of 18.5 kDa MBP is involved in SH3-domain binding [8,11,22,23]. We have referred to the longer S72–S107 peptide previously as the α2-peptide because it comprises the second of three amphipathic α-helical segments involved in membrane-association [39,40]. In our first comprehensive study of this interaction, we assumed that the proline-rich region containing a single P–x–x–P consensus SH3-ligand formed the common PPII conformation, and performed *in silico* rigid-body docking experiments of a model of the (T92–R104) segment with the crystallographic structure of Fyn-SH3 [8]. The results showed that this region canonically interacts with the SH3-domain binding site via salt bridges and cation-*π* interactions, and that modifications such as phosphorylation caused their relative orientations to adapt to minimize the docked energy. At the time, this assumption of a PPII conformation in MBP bound to Fyn-SH3 seemed quite reasonable for this type of protein [30,41], especially given that we had demonstrated transient PPII structuring in both free and dodecylphosphocholine-associated 18.5 kDa MBP (full-length protein) by collecting circular dichroism spectra at variable temperatures [8,14]. The CD experiments could not define which regions of the protein adopted PPII, as they reported only global secondary structure. However, in later solution NMR and molecular dynamic simulation experiments involving the α2-peptide (S72–S107), we found that there is little tendency for the unbound proline-rich region to adopt a PPII conformation in an aqueous environment, leading us to conclude that such a conformation is either unnecessary for interaction with SH3-domains or is induced upon binding [23]. The formation of PPII structure *per se* is one of the hypotheses that we explore here.

In another study, we found that co-transfection of an immortalized early oligodendrocyte cell line (N19-cells), with constitutively-active Fyn kinase and 18.5 kDa MBP produced a cell morphology not obtained from transfections of either construct alone. Variants of 18.5 kDa MBP with pseudo-phosphorylation at T92 and T95, or P-to-G substitutions within the proline-rich region, resulted in cell morphology that was less pronounced than for unmodified 18.5 kDa MBP. These results support the conjecture that this region is, indeed, essential for the interaction of the two proteins and maintenance of phenotype *in vivo* [10]. A full thermodynamic characterization of this interaction, and the effects of these substitutions, was elusive, however. ITC (isothermal titration calorimetry) experiments involving the hexa-histidine-tagged full-length MBP and the 9.3 kDa Fyn–SH3 domain produced distorted binding curves due to aggregation, although we were able to discern clear heats of interaction and differences due to pseudo-phosphorylation [10]. These problems related to aggregation are alleviated using smaller, untagged peptides of MBP. Still, it has proven non-trivial to characterize the association of simple peptides comprising just the proline-rich region with the 9.3 kDa Fyn–SH3 domain *in vitro* [10,11]. For example, the segment (F86–G103) yielded only small endothermic heats in ITC experiments, a result that suggests non-canonical interaction in contrast to what was assumed in our first *in silico* molecular docking experiments. It should be noted that subsequent solution NMR spectroscopy of the α2-peptide confirmed that the proline-rich segment participates directly in the interaction with Fyn–SH3 *in vitro*, consistent with the cell-transfection studies with variants [11]. However, this result does not rule out the possibility that other regions within the protein may also participate, especially in the context of a possible non-canonical binding mechanism.

In the present study, our objectives were to identify the regions of MBP essential for its physiological interaction with Fyn–SH3, and to observe the structural changes and residues involved in the interface. We first used *in cellula* imaging to define the minimal segments of MBP required for interaction with Fyn–SH3 as demonstrated by phenotype, followed by solution NMR spectroscopy of a recombinant construct of this region and the Fyn–SH3 domain. This new peptide spanned residues (S38–S107) and is referred to here as the *xα*2-peptide, because it is an extension of the previously-studied α2-peptide spanning residues (S72–S107) [11,40]. This ‘divide and conquer’ strategy was necessary to minimize the aggregation effects observed when full-length MBP and Fyn–SH3 are titrated, allowing a more effective observation, by 2D and 3D NMR spectroscopy, of the chemical shifts of the residues involved in binding. Using the *xα*2-peptide, we performed NMR titration experiments that enabled us to define the *K*_D_ of interaction with Fyn–SH3, and we have also identified a region within MBP, upstream of the proline-rich region, that participates in binding both *in vitro* and *in cellula*. We demonstrate further that the *xα*2-peptide-Fyn–SH3 complex is dynamic, and there is little evidence of binding-induced periodic secondary structure formation in the peptide, suggesting that this may be a new example of a ‘fuzzy’ MBP complex.

## EXPERIMENTAL

### Plasmid construction for transfection of N19-oligodendroglial cells

The previously described plasmids coding for an RFP (red fluorescent protein)-tagged version of the unmodified charge component of 18.5 kDa MBP, possessing a 3′-untranslated region (UTR, within the plasmid pERFP-C1-rmMBPC1-UTR), were used here as template DNA from which the N-terminal truncation constructs were made [42]. The MBP variants had RFP fused to the amino terminus, and the plasmid encoded a 3′UTR to facilitate proper trafficking of the mRNA to the cell periphery [42,43]. PCR amplifications were performed using a BioRad thermal cycler PCR system and *Taq* polymerase (Invitrogen Life Technologies) with the following cycling parameters: initial denaturing temperature of 95°C for 5 min, followed by 25 cycles of 95°C for 30 s, 60°C for 30 s, 72°C for 2 min, followed by a final 4°C hold. The 18.5 kDa MBP, N-terminal variants were cloned into the pERFP-C1 vector using *Bsp*EI and *Bcl*I restriction sites that encompassed the previously cloned 3′UTR signal. Primers that were used to introduce the *Bsp*EI and *Bcl*I restriction sites for constructs are provided in Supplementary Table S1. Amplified products were digested with *Bsp*EI and *Bcl*I, and were ligated into the pERFP-C1 vector. Positive clones were confirmed by sequencing (Laboratory Services Division, University of Guelph).

### Cell culture and transfection

For transfection experiments, DNA was extracted using the PureLink HiPure Plasmid Purification kit (Invitrogen Life Technologies. Other reagents used for these studies were purchased from either Thermofisher Scientific or Sigma-Aldrich unless otherwise stated. Cell lines, cell culture and transfection were performed as previously described [42]. Tissue culture reagents were purchased from Gibco/Invitrogen. The FuGene HD transfection reagent was purchased from Roche Diagnostics. The immortalized N19-glial cell cultures [44,45] were grown in DMEM (Dulbecco's modified Eagle's medium) high-glucose media supplemented with 10% (w/v) FBS and 1% penicillin/streptomycin, and cultured in 10 cm plates at 34°C/5% (v/v) CO_2_. At 70–80% confluency (4–7 days), cells were detached using 0.25% (w/v) trypsin for 5 min. Using a haemocytometer, live cells were counted, plated at a density of 0.5 × 10^6^ cells/ml, and grown overnight in preparation for transfection experiments. The following day, the cells were co-transfected using 100 μl serum-free media, 2 μg of MBP plasmid DNA, 1 μg of constitutively-active Fyn (p59Fyn–Y527F) plasmid DNA, and 4 μl of FuGene HD (Roche Diagnostics). The DNA was allowed to complex for 5 min at room temperature, and was directly added to cells following incubation. Cells were cultured for an additional 48 h at 34°C prior to treatment, fixation or immunoprocessing.

### Immunofluorescence microscopy and image analyses

Following protein expression, cells were directly fixed using 4% (v/v) formaldehyde solution in PBS (137 mM NaCl, 2.7 mM KCl, 10 mM Na_2_HPO_4_, 1.8 mM KH_2_PO_4_, pH 7.4) for 15 min with gentle rocking. Samples requiring immunoprocessing were permeabilized using 0.1% (v/v) Triton X-100 for 20 min, and were subsequently washed once with 1 ml of PBS. Slides were blocked for 1 h using 10% NGS (normal goat serum) and, following this incubation, the primary antibody (Anti-Fyn Rabbit pAb, Cell Signaling Catalog No. 4023) was added and incubated for an additional hour to confirm that cultures were expressing exogenous Fyn kinase. The slides were then washed three times with 1 ml PBS, and the secondary antibody (1:400 dilution) was applied for 20 min. Once again, the slides were washed four times with 1 ml of PBS, and were mounted using ProLong Gold AntiFade reagent containing DAPI (4′,6-diamidino-2-phenylindole; Invitrogen). Slides were viewed using a Leica epifluorescence microscope (DMRA2) and images were processed and analysed using ImageJ software (National Institutes of Health (http://rsb.info.nih.gov/ij/), and were compiled using Adobe Photoshop CS3.

### Statistical analyses of N19-cell proliferation

For statistical analysis, a set of 150 transfected N19-cells expressing MBP and constitutively active Fyn kinase at an average level were selected, and the extent of third-degree process branching was scored. Phenotypes from two separate experiments were evaluated on different days, producing two sample sets (*s*=2), each examining 150 cells (*n*=150) for each N-terminal variant. A paired *t* test (*P* =  0.05) was used to determine that the duplicate sample sets did not differ significantly. Afterwards, the recorded measurements from each duplicate were grouped into a larger sample set of *n*=300. The N19-cells from each experiment were then compared with one another using an ANOVA table (*P* =  0.05), and the means of each variant and the S.E.M. for each trait were determined. The difference in means was further analysed using the Tukey means comparison test (*P* =  0.05) to determine which variants were significantly different from each other for each trait measured.

### Untagged 18.5 kDa MBP Purification

The recombinant murine 18.5 kDa isoform of MBP, with no purification tags (168 residues), was expressed and purified as previously described [46].

### The *xα*2(S38–S107) peptide purification

The *xα*2(S38–S107) peptide was constructed and expressed in M9 minimal media as discussed previously [11,23,40], and supplemented with ^15^N-labeled NH_4_Cl (for all NMR experiments) and ^13^C-labeled glucose (for NMR assignment experiments only) provided by Cambridge Isotope Laboratories. This peptide was purified in a similar fashion to previously studied MBP α-peptides [*ibid*.] with a few modifications to the protocol. Following a native lysis procedure, immobilized metal affinity chromatography (using Ni-NTA resin) was used to purify the SUMO (small ubiquitin-related modifier)-tagged peptide. In the subsequent step, SUMO protease I (UlpI, kindly provided by C. D. Lima, Sloan-Kettering Memorial Institute, New York, NY) was used as discussed previously to remove the SUMO tag and yield untagged peptide. Afterwards, the buffer was exchanged to a 50 mM glycine buffer at pH 10 to stop the protease activity and to prepare for cation exchange chromatography. The fractions containing the peptide were then further purified and desalted by reversed-phase HPLC. The final product was lyophilized completely on a Labconco FreeZone freeze-dryer (Fisher Scientific). To confirm the molecular mass, we submitted a purified peptide sample to MALDI-TOF (matrix-assisted laser desorption/ionization time-of-flight) mass spectrometry (Advanced Analysis Centre, University of Guelph). The average purification yield was approximately 5 mg fully labelled, or unlabeled, peptide per 1 litre of culture, at almost 99% purity.

### Fyn–SH3-domain purification

The plasmid encoding the *Gallus gallus* (chicken) SH3-domain of Fyn was a generous gift of Dr Alan Davidson, University of Toronto, and was expressed and purified as previously described [47]. The construct comprised residues 85–142 of Fyn, a FLAG epitope and a His_6_-tag at the C-terminus, and a short N-terminal tail.

### Solution NMR Spectroscopy

The ^13^C-labelled glucose, ^15^N-labelled NH_4_Cl, and D_2_O were purchased from Cambridge Isotope Laboratories. All the NMR experiments on the *xα*2-peptide were recorded on a Bruker Avance spectrometer operating at a Larmor frequency of 600.1 MHz. The titrations were carried out with 3 mg of ^15^N-labelled *xα*2(S38–S107) being dissolved in a Hepes buffer [20 mM Hepes, 100 mM NaCl and 10% ^2^H_2_O, pH 7.4] to a total volume of 500 μl (final concentration 0.8 mM). The Fyn–SH3 domain peptide was dissolved in the same buffer and titrated into the *xα*2-peptide solution to observe the chemical shift changes of the *xα*2-peptide in 2D ^15^N-HSQC spectra at different molar ratios (1:0, 1:0.1, 1:0.2, 1:0.4, 1:0.5, 1:0.6, 1:0.7, 1:0.8, 1:1.0, 1:1.1, 1:1.2).

Fitting of titration data to obtain the dissociation constant *K*_D_ was performed using the OriginPro software package, version 8.0 (OriginLab). The chemical shift perturbations observed at each of the molar ratios were quantified by the following equation [48,49]:

(1) $$\Delta \delta =\sqrt{\Delta \delta {{(}^{1}H)}^{2}+\frac{1}{4}\Delta \delta {{(}^{15}N)}^{2}}$$

The change in chemical shifts was converted to fractional saturation, and plotted against the molar ratio of Fyn–SH3 to *xα*2-peptide, or against the total Fyn–SH3 concentration for the purpose of fitting. The saturation curves were fit with the following equation:

(2) $$f=\frac{{\mathrm{Fyn}}_{t}+n\cdot x\alpha 2_{t}+K_{D}-\sqrt{{\left({\mathrm{Fyn}}_{t}+n\cdot x\alpha 2_{t}+K_{D}\right)}^{2}-4\cdot {\mathrm{Fyn}}_{t}\cdot n\cdot x\alpha 2_{t}}}{2\cdot n\cdot x\alpha 2_{t}}$$

where ‘*Fyn_t_*’ is the total concentration of Fyn–SH3 domain, ‘*xα*2*_t_*’ is the concentration of *xα*2-peptide, ‘*n*’ is the stoichiometry, and ‘*K*_D_’ is the dissociation constant. Equation (2) allowed calculation of the stoichiometry of interaction and estimation of the dissociation constant *K*_D_ from the chemical shift perturbations.

The acquisition parameters for these experiments can be found in Supplementary Table S2. The assignment strategy for the *xα*2(S38–S107) peptide was the same as used previously [11,23]. Collection of a 2D ^15^N-HSQC experiment was followed by several triple-resonance experiment pairs: (a) HNCO/HN(CA)CO to assign the amide proton (^1^H_N_[*i*]) and amide nitrogen (^15^N[*i*]) of each spin system, their associate carboxyl carbons (^13^C’[*i*]), and the carboxyl carbon of the residue preceding it (^13^C’[*i-1*]); (b) CBCA(CO)NH/HNCACB to assign the C_α_ and the C_β_ atom of each residue and the residue preceding it; and (c) HACAN to identify prolyl residues [50]. Water suppression was achieved using the double-pulsed field gradient spin echo technique (excitation sculpting) with the carrier frequency set to the water ^1^H signal.

The ^1^H chemical shifts were referenced directly to DSS (2,2-dimethylsilapentane-5-sulfonic acid) in an external sample, and the ^13^C and ^15^N chemical shifts were referenced indirectly. All spectra were processed using NMRPipe [51]. All free induction decays were zero-filled, and apodized using a shifted, squared sinusoidal bell function prior to Fourier transformation and subsequent phase-correction. The ^1^H–^15^N–HSQC spectrum was zero-filled up to 2048 and 4096 complex points along *F*_1_ and *F*_2_, respectively. The HN(CA)CO, HNCACB, and their complementary spectra were zero-filled up to 256, 256 and 2048 complex points along *F*_1_, *F*_2_ and *F*_3_, respectively. The HACAN and HCCH spectrum were zero-filled up to 256, 256 and 2048 complex points along *F*_1_, *F*_2_ and *F*_3_, respectively. The ^1^H–^13^C–HSQC spectrum was zero-filled up to 4096 complex points along each of *F*_1_ and *F*_2_. Spin systems were then assigned using Computer Assisted Resonance Assignment (CARA, version 1.8.4) [52], modules contained in the CARA software package (www.nmr.ch), and a collection of in-house scripts that have been previously described. Spin system creation strategies used by the scripts, as well as spin selection strategies, have been described previously [11,23,53]. Any spin systems not identified by the scripts were selected manually. Sequence-specific connectivity was obtained manually by iterative trials.

The chemical shifts assignments obtained (H_α_, C_α_, C_β_, C′, N and H_N_ specifically) were used to predict secondary structure. The Vendruscolo Laboratory's δ2D software was used, which implements an algorithm designed to calculate the probability that any amino acid in a primarily disordered protein has a particular secondary structure conformation, based on the chemical shift assignments [54].

The ^1^H and ^13^C chemical shifts of this extended *xα*2-peptide have been deposited in the BioMagResBank (www.bmrb.wisc.edu) with accession numbers 19948 (uncomplexed) and 19949 (with unlabelled Fyn–SH3 at 1:1 molar ratio), for comparison with previously published and deposited data for the shorter α2-peptide (BMRB 18520 and 19972, respectively) [11].

### ^15^N Relaxation measurements

The relaxation parameters of ^15^N were measured using standard approaches [55–57]. The relaxation rates were determined from the decay of intensity of each ^15^N–^1^H cross-peak in a series of spectra with increasing time delays. The *R*_1_ measurements were conducted with relaxation delays of 1.6, 50, 100, 200, 300, 400, 800, 1000 and 1200 ms. The transverse relaxation constant *R*_2_ was done with CPMG (Carr–Purcell–Meiboom–Gill) delays of 64, 128, 160, 192, 224 and 250 ms. The relaxation constants and experimental errors were extracted by exponential curve fitting of the peak heights using the built-in option of SPARKY [T.D. Goddard and D.G. Kneller, SPARKY 3, University of California, San Francisco] as discussed previously [53].

The heteronuclear steady-state ^1^H–^15^N–NOE (nuclear Overhauser effect) intensities were obtained from the ratio *I_NOE_*/*I_NONOE_* of peak heights in the NOE spectra with and without proton saturation, respectively. The spectra were collected using a standard experiment [55], and uncertainty in measuring NOE intensities was carried out as described previously [53].

## RESULTS

### An 18.5 kDa MBP segment upstream of the primary SH3-ligand is involved in interaction

We reasoned on the basis of our previous experience that constructing a smaller peptide of MBP that could still interact in a mode that is representative of the full-length protein could potentially ameliorate difficulties with aggregation, particularly at the relatively high protein concentrations required for ITC and NMR spectroscopy [10,11,58]. In constructing this peptide, we first used *in cellula* imaging to define the minimal segments of MBP required for interaction with Fyn–SH3 as assessed by morphological phenotype of the immortalized N19-cell line, representing an early-developmental oligodendrocyte [10,44,45].

**Figure 2:**
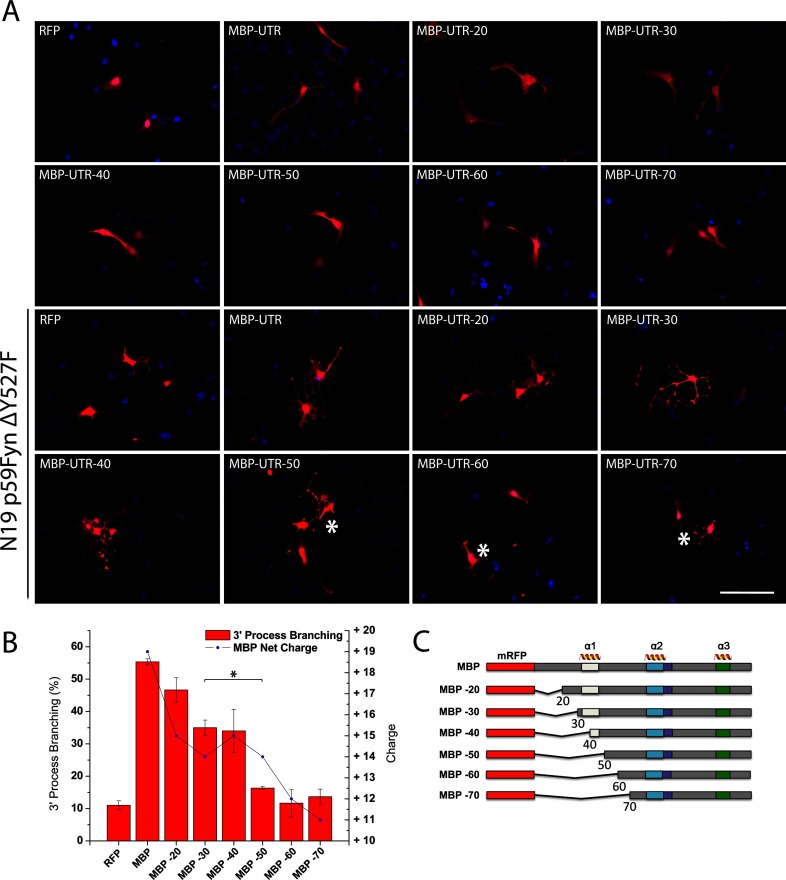
The 18.5-kDa isoform of MBP requires regions upstream of the poly-proline target to interact with Fyn kinase in N19-cells (**A**) Fluorescence micrographs of cultured N19-cells, 2 days post-transfection, overexpressing wild-type and N-terminal deletion variants of RFP-tagged MBP (red, schematized in panel **C**), co-expressing constitutively active Fyn (p59Fyn-Y527F) (not shown). Fyn was immunostained and detected using secondary Alexa594-conjugated antibodies, along with nuclei counterstained with DAPI (blue). The N19-OLGs co-expressing constitutively-active Fyn, along with MBP, demonstrated increases in third degree branching complexity, as previously reported by us [10]. The N-terminal deletion mutations of MBP showed a continuous decline in the number of cells exhibiting increased branching complexity, which was attributable to the severity of the N-terminal deletion. The most notable decreases in branching complexity were observed for MBP-50, MBP-60 and MBP-70, mutations that were structurally devoid of the predicted N-terminal amphipathic α-helix that was previously shown to polymerize and bundle actin, and bind to Ca^2+^–CaM [40,59]. (**B**) Statistical analysis demonstrating the extent of third degree process branching of N19-cells transfected with MBP or N-terminal deletion variants when co-expressed with constitutively active Fyn (p59Fyn-Y527F) (red histograms). The overall net-charge of the 18.5-kDa MBP variant was also plotted for each experiment (line, blue dots). Significant decrease in process branching is likely caused by loss of the key N-terminal structural domain of MBP, as opposed to a charge-dependent inhibition as that is particularity evident when comparing MBP-30 and MBP-50 (asterisk). (**C**) Schematic of the full-length and N-terminal deletion variants of RFP-tagged MBP with amphipathic α-helical segments marked above. Bar=50 μm.

To identify first the region of MBP essential for the interaction with Fyn–SH3, we generated a series of N-terminal truncation mutants (−20, −30, …, −70), all tagged with a monomeric RFP at the N-terminus, and a C-terminal untranslated region on the mRNA for proper targeting within the cell (Figure 2C and Supplementary Table S1) [42]. We conducted the truncations at the N-terminus specifically. The reason is that previous ITC experiments with N- and C-terminal deletion variants of MBP, both containing the proline-rich region, demonstrated that the binding between MBP and Fyn is non-specific in the absence of the 56 N-terminal amino acids, but is not affected by the absence of 63 C-terminal residues [10]. Here, when each of these constructs was transfected into the N19-cells, the cells exhibited a more extended morphology (Figure 2A) than when they were transfected with RFP alone. The cells that were co-transfected with both constitutively-active Fyn and full-length 18.5 kDa MBP exhibited third-degree (tertiary) branching as observed previously [10] that would not be present with transfection of either protein alone (Figures 2A and 2B). As the MBP truncations became more pronounced, the percentage of the transfected cells that exhibited this phenotype decreased. The most significant drop in the population of branched N19-cells was between the −40 and the −50 residue truncation of MBP. This drop was independent of the net charge of the constructs, since both the −50 and the −30 truncations have the same net charge at neutral pH (+14) (Figure 2B). These observations suggest that the minimum required portion of the N-terminus for maintaining the observed phenotype, and potentially SH3-binding, begins between residues G40 and P50. This region coincides with the end of the first amphipathic α-helix that is observed when MBP is in membranous or membrane-mimetic environments (T33–D46) (Figure 1B) [39,40,59].

Based on these results in N19-cell culture, we generated a new MBP-peptide, spanning residues (S38–S107), and referred to here as the *xα*2-peptide (Figure 1C and Supplementary Figure S1) [11,40]. We confirmed that this new peptide did not aggregate when mixed with the Fyn–SH3 domain, and performed simple glutaraldehyde cross-linking experiments that demonstrated that it interacts with Fyn–SH3 *in vitro* (Supplementary Figure S2) with a stoichiometry of 1 to 1. We then performed an NMR titration experiment with the *xα*2-peptide and Fyn–SH3 in order to probe their interaction in greater detail.

### NMR spectroscopy demonstrates that the *xα*2-peptide and Fyn–SH3 interact with a micromolar *K*_D_ and saturation at a 1:1 molar ratio

**Figure 3:**
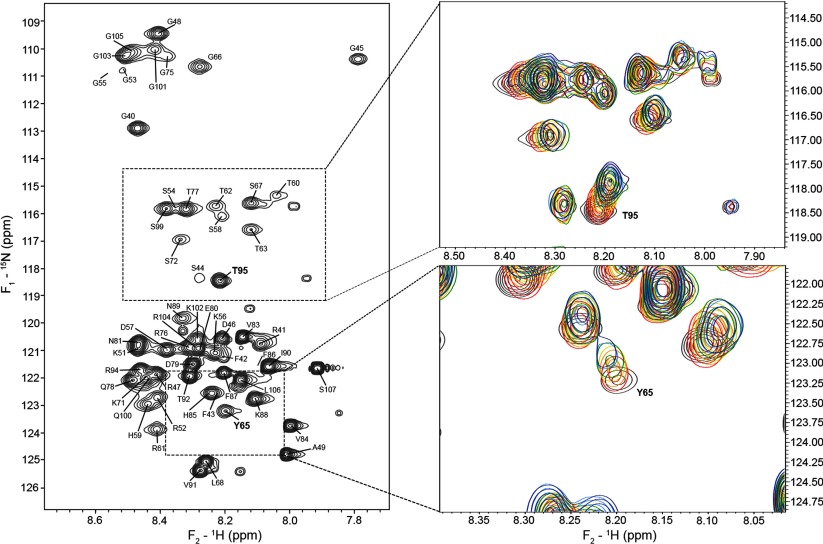
Titration of unlabeled Fyn-SH3 domain (9.3 kDa) into ^15^N-labelled *xα*2-peptide (7.6 kDa, residues S38–S107) The ^15^N-HSQC of the chemical shift perturbations of the *xα*2-peptide is shown in grey in the left-hand panel. The insets and the right-hand panel show the chemical shift perturbations of the peptide upon titration with Fyn–SH3 at molar ratios (Fyn–SH3/*xα*2-peptide) of 0 (grey), 0.2 (red), 0.4 (orange), 0.6 (yellow), 0.8 (green), 1 (blue), 1:1.2 (purple).

Previously, solution NMR spectroscopy was used to show that the shorter α2-peptide interacted with Fyn–SH3 as evidenced by peak shifts in the HSQC spectrum [11], and a more detailed study was performed here with the longer *xα*2-peptide (Figure S1). One of the major challenges that needed to be addressed when studying MBP with Fyn–SH3 in aqueous solution was *in vitro* aggregation. By reducing the size of the peptide to a region of MBP that was physiologically relevant to the interaction, we were successful in eliminating aggregation, enabling us to use solution NMR spectroscopy (acquisition parameters shown in Supplementary Table S2). The molecular mass of the 1:1 complex would be ~16.9 kDa (*xα*2-peptide is 7.6 kDa, Fyn–SH3 domain is 9.3 kDa). As expected for a segment of the intrinsically disordered MBP, the **xα**2(S38–S107) peptide shows poor dispersion in the proton dimension (7.7–8.6 ppm) of the ^15^N-HSQC spectrum (Figure 3). By combining chemical shifts obtained in a series of 3D experiments, we were able to overcome the chemical-shift degeneracy inherent to the peptide and assign most of the backbone residues. The ^1^H and ^13^C chemical shifts of this extended *xα*2-peptide have been deposited in the BioMagResBank (www.bmrb.wisc.edu) with accession numbers 19948 (uncomplexed) and 19949 (with unlabeled Fyn–SH3 at 1:1 molar ratio), for comparison with previously published and deposited data for the shorter α2-peptide (BMRB 18520 and 19972, respectively) [11].

Analysis of the chemical shifts of the *xα*2-peptide (7.6 kDa), using the δ2D method of Camilloni *et al*. designed for disordered proteins [54], revealed its secondary structure propensities. This analysis demonstrated that the peptide is predominantly random coil (82.8%) and there is very low probability to form any other ordered secondary structural elements (Supplementary Figure S3). The result of this analysis is unsurprising as any peptide of MBP would be expected to sample a variety of extended conformations in an aqueous environment, due to it being intrinsically disordered and highly cationic [11,23,40]. We have previously reported that the conformations which are sampled by MBP generally have very few ordered secondary structure elements present in an aqueous environment [39,60]. In performing a similar δ2D analysis of the *xα*2-peptide bound to Fyn–SH3 (9.3 kDa), it was somewhat surprising that we found no significant change in secondary structure propensity with an overall random coil content of 83.5%.

**Figure 4:**
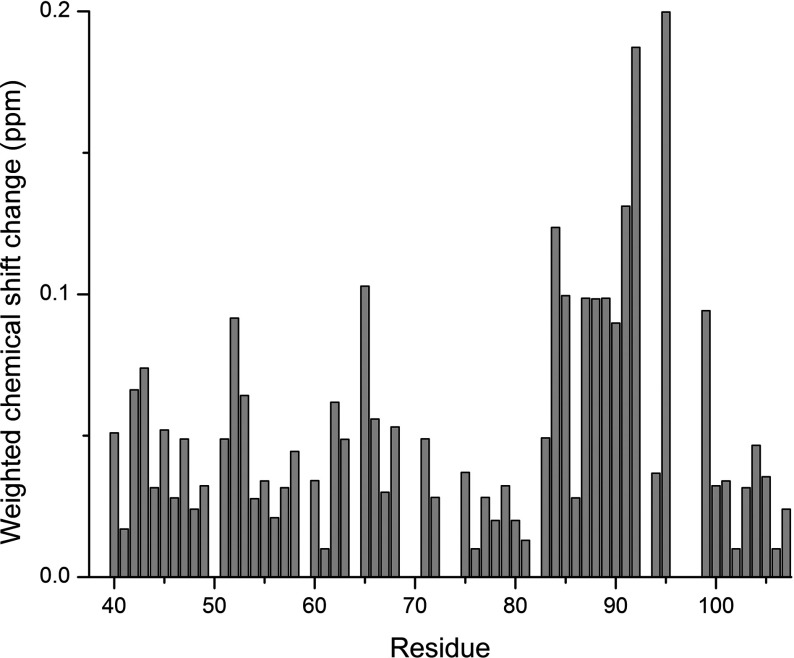
Weighted chemical shift change per residue of the *xα*2-peptide upon titration of Fyn–SH3 The HN and N chemical shifts were weighted and averaged for all assigned non-prolyl residues of the *xα*2-peptide alone, and at 1:1 Fyn–SH3, by using Equation (1).

**Figure 5:**
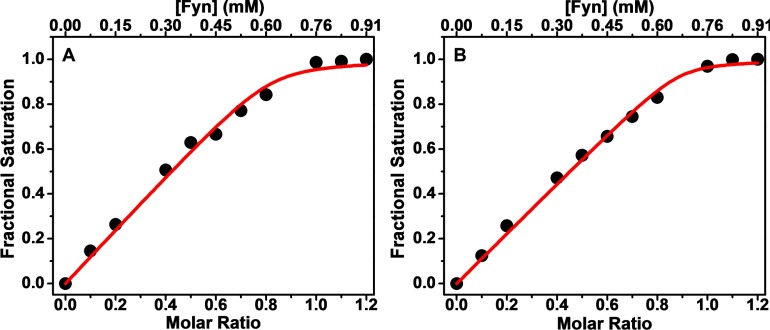
Chemical shift changes of the (A) Y65 and (B) T95 peaks of the *xα*2-peptide upon titration of Fyn-SH3 Data were fit to a fractional saturation model [Equation (2)] to obtain the value of *K*_D_ and the stoichiometry (Supplementary Table S3).

The observed chemical shifts of the *xα*2-peptide were systematically perturbed upon titration with the Fyn–SH3 domain, thus further confirming their interaction (Figure 4). The Y65 and T95 chemical shifts were used to quantify the fractional saturation of chemical shift change [Equation (1)], which was plotted versus total concentration of Fyn–SH3, showing that saturation was achieved at the 1:1 molar ratio (Figure 5). Data fitting to a fractional saturation equation yielded *K*_D_ values of 7.6±4.6 and 4.0±2.5 μM with a stoichiometry of 1 for the Y65 and T95 data, respectively [Equation (2)]. It is important to note that these *K*_D_ values are only estimates, and much lower protein concentrations would be required to obtain more reliable figures [61]. Here, the NMR experiments were of necessity done at relatively high protein concentrations to obtain an adequate signal.

We also investigated the interaction of MBP and Fyn–SH3 with ITC at 22 and 37°C. In previous calorimetry experiments, thermodynamic parameters of the interaction of a hexa-histidine-tagged recombinant 18.5 kDa MBP construct Fyn–SH3 could not be extracted due to aggregation [10]. Here, using a new untagged construct for 18.5 kDa MBP [46] and a more sensitive calorimeter, we observed a significant reduction in aggregation and only the first few points of the binding curve appeared anomalous (Supplementary Figure S4). However, even a small presence of aggregates means that only apparent or estimated *K*_D_ and Δ*H* values can be extracted from ITC data fitting rather than true thermodynamic values (Supplementary Figure S4 and Supplementary Table S3). The *apparent K*_D_ values, derived from these ITC experiments, suggest a low micromolar binding constant, in general agreement with estimates obtained by NMR spectroscopy for the *xα*2-peptide and are wholly consistent with the ranges reported in the literature (e.g., [34]). We also performed ITC experiments with the *xα*2-peptide and Fyn–SH3 at 22°C and 37°C; however, we detected minimal measurable heat changes (not shown) despite the fact that the *in cellula* data (Figure 2), *in vitro* cross-linking experiments (Figure S2) and NMR experiments (Figures 3–5), demonstrate a clear and measurable interaction. The lack of heats of interactions in ITC experiments involving the *xα*2-peptide, in contrast to the full-length protein, could be due to the absence of long-range conformational changes in the shorter peptide that may occur upon binding of Fyn–SH3 to full-length 18.5 kDa MBP. These conformational changes may contribute to measured heat, but may not be critical to the interaction of MBP and Fyn–SH3.

### NMR relaxation and NOE measurements confirms that a segment, N-terminal to the canonical ligand in MBP is involved in Fyn–SH3 interaction

**Figure 6:**
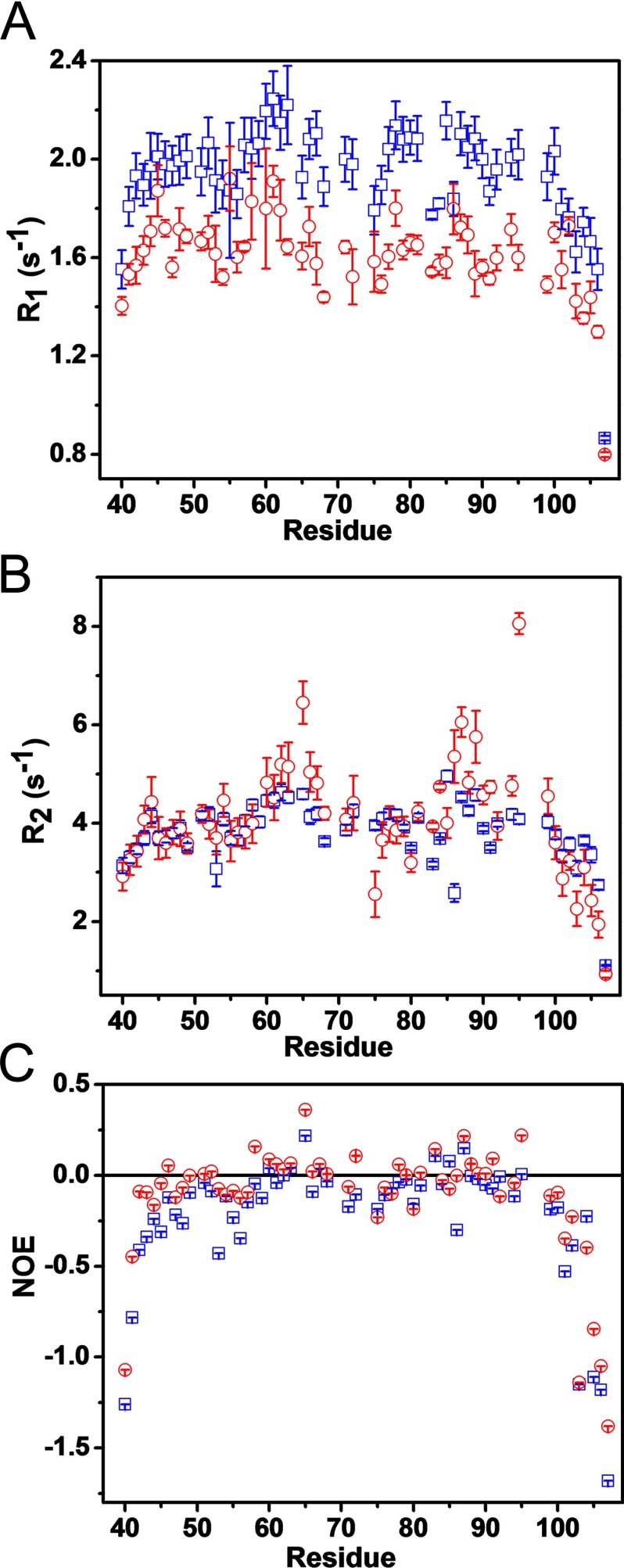
Relaxation (^15^N) and NOE measurements of interactions of *xα*2-peptide with Fyn–SH3 (**A**) Longitudinal (*R*_1_) and (**B**) transverse (*R*_2_) relaxation measurements of the *xα*2-peptide alone (blue squares), and in the presence of Fyn–SH3 (red circles). Relaxation measurements (^15^N) of the *xα*2-peptide alone and in the presence of Fyn–SH3, measured using standard approaches [55–57]. The relaxation constants and experimental errors were extracted by exponential curve fitting of the peak heights using the built-in option of SPARKY [T.D. Goddard and D.G. Kneller, SPARKY 3, University of California, San Francisco] as discussed previously [53]. (**C**) Heteronuclear NOE measurements of the *xα*2-peptide alone (blue squares), and in the presence of Fyn–SH3 (red circles). The steady-state ^1^H-^15^N NOE intensities were obtained from the ratio *I_NOE_*/*I_NONOE_* of peak heights in the NOE spectra with and without proton saturation, respectively. The spectra were collected using a standard experiment [55], and uncertainty in measuring NOE intensities was carried out as described previously [53].

The longitudinal relaxation (*R*_1_) measurements of the peptide alone revealed a featureless profile with relaxation rates ranging from 0.866 to 2.2 s^−1^, with an average value of 1.94 s^−1^ (Figure 6A). In the presence of Fyn–SH3, the majority of the *R*_1_ values obtained are significantly lower, but still the profile is featureless, with relaxation rates ranging from 0.8 to 1.92 s^−1^, with an average value of 1.6 s^−1^. This result indicates that the overall tumbling of the peptide backbone has lowered.

The transverse relaxation (*R*_2_) values obtained for the peptide alone were more varied and sequence-dependent, and ranged between 1.1 and 5.0 s^−1^ (Figure 6B). In the presence of Fyn–SH3, the *R*_2_ values ranged between 0.92 and 8.06 s^−1^, with regions (T62–L68) and (V83–S99, excepting H85 and T92) showing increased transverse relaxation rates. The highest transverse relaxation rate found was for T95, the residue immediately preceding the tri-proline repeat in the proline-rich region of MBP. The *R*_2_ rates obtained in the presence and the absence of Fyn–SH3 highlighted a change in two distinct regions of the peptide: (T62–L68), and (V83–S99) (with H85 and T92 as exceptions) (Figure 6B). These results demonstrate further that both of these regions of the *xα*2-peptide are involved in the interaction with Fyn–SH3, consistent with the chemical shift perturbations.

The steady-state NOEs of both *xα*2-peptide alone, and complexed with Fyn–SH3, were measured to analyse the dynamics of the peptide at the picosecond-nanosecond timescale (Figure 6C). The NOEs of the *xα*2-peptide alone and with Fyn–SH3 exhibit similarly featureless, flattened bell-shaped variation throughout the length of the peptide, with most of the values being negative. In the case of *xα*2-peptide alone, the NOEs range from −1.68 to 0.22, with an average of ~ −0.2. In the presence of Fyn–SH3, the NOEs increased slightly, ranging from −1.38 to 0.36, averaging ~ −0.1. Negative NOEs are suggestive of dynamic motions traditional of IDPs on the picosecond to nanosecond timescale [53], suggesting that *xα*2-peptide has more restricted backbone dynamic motions in the presence of Fyn–SH3. The NOEs observed in both cases are still relatively high for completely dynamic IDPs of this size [62], suggesting there is some restriction to the dynamics in both cases. The highest NOE value recorded was for Y65, suggesting that its motion is restricted in the presence of Fyn–SH3, and T95 also shows an increase from a near-zero value to a positive NOE, in agreement with the results observed from the transverse relaxation experiments.

## DISCUSSION

### The MBP–Fyn interaction is non-canonical

The interaction of 18.5 kDa MBP with the SH3–domain of Fyn is clearly direct and specific, yet has defied easy structural description. The central segment of 18.5 kDa MBP is highly-conserved and comprises an amphipathic membrane-associated α-helix (maximally residues V83–T92), immediately adjacent to the putative P–x–x–P consensus SH3-ligand (P93–R94–T95–P96) and a proline-rich segment (P96–P97–P98). The α-helical segment was shown by NMR spectroscopy to be transient in aqueous solution, yet it is stabilized by membrane-mimetic solvents and definitively forms on myelin-mimetic membranes [39,63,64]. Transient poly-proline type II structure in the full-length protein could also be demonstrated by variable-temperature CD spectroscopy in aqueous solution and in dodecylphosphocholine micelles [8,14]. A molecular model of this central segment of MBP containing adjacent, preformed α-helix and poly-proline type II structural elements could be docked to the crystallographic structure of Fyn–SH3 *in silico*, and could be interpreted straightforwardly [8]. However, ITC of peptide fragments comprising this central segment of MBP did not yield classic binding isotherms to Fyn–SH3 [10], and the interaction could only be demonstrated *in vitro* by NMR spectroscopy [11]. The interaction of these two proteins is thus non-canonical, necessitating the *in vitro* NMR and other studies performed here to define better the interface of 18.5 kDa MBP that interacts with the Fyn–SH3 domain. In their excellent review, Uversky and Dunker discuss the varieties of coupled folding and binding of IDPs [16]. In this context here, the questions addressed were whether the putative SH3–ligand of MBP is sufficient for its interaction with Fyn–SH3, and if a poly-proline type II structure is induced and/or fixed in the complex.

The involvement of the (T62–L68) region of 18.5 kDa MBP in its interaction with the Fyn–SH3 domain is a new finding. This segment is located over 20 residues upstream from the proline-rich region of MBP between the first and the second amphipathic α-helices in a region that has not shown very much secondary structure in any of the molecular environments studied previously (see Figure 1) [39,60]. We also know from previous studies that this region is not absolutely critical for interaction, since the shorter α2-peptide of MBP (S72–S107), which lacks this segment, is still capable of binding Fyn–SH3 as shown by chemical shift perturbations localized in the proline-rich region [11]. However, saturation was not achieved even at a 1:1.2 molar ratio for this shorter α2-peptide (Supplementary Figure S5), suggesting that the presence of the T62–L68 segment is important for achieving binding saturation at a 1:1 molar ratio (Figure 5), as well as being crucial for maintaining the branched phenotype of the N19-cells (Figures 2A and 2B).

### The interaction of 18.5 kDa MBP with Fyn–SH3 involves long-range interactions with no discernible induced secondary structure and is consistent with a fuzzy complex

The interactions of SH3–domains with their ligands are modulated by residues flanking, or within, the consensus sequence of the ligand [34,36,65,66]. In the case of MBP, not only do residues adjacent to the binding site influence the interaction, but an additional portion of MBP that is over 20 residues away is also involved. Although there is little to no change in ordered secondary structure observed between the free and bound states, 18.5 kDa MBP (being an IDP) can easily undergo a global conformational change which would facilitate interactions with residues and/or regions that are far away from the canonical binding site.

Fuzzy complexes comprise a group of protein–protein interactions within IDPs that do not follow straightforward disorder-to-order transitions upon binding with their ligand [18,19]. Despite being directly involved in binding, the intrinsically disordered regions retain their dynamic nature and cannot be described by one conformational state when bound [18]. This phenomenon has been observed in a variety of different proteins, including 18.5 kDa MBP [21,67–70]. The SH3–domain interactions fall into this category, but the SH3–ligands usually exhibit an increase in their secondary structure propensity, such as a poly-proline type II conformation [41,71]. In the unbound state, these SH3–ligands seem to exhibit pre-existing conformations transiently. These residual structural elements observed in the unbound state aid in the transition of ensembles to the bound state.

Interestingly, here, the *xα*2-peptide showed minimal secondary structure propensities in the unbound state, which did not change upon Fyn–SH3 binding. Despite having two distinct regions involved in SH3–domain binding, the structural ensembles of the *xα*2-peptide seem to be minimally restrained at the secondary structural level. In contrast, the observed broadening of two separate groups of amide chemical shifts suggests that in the unbound state, MBP transitions to an ensemble of structures that have a more compact global fold, without restricting the secondary structure dynamics. The literature reports many exceptions to the PPII target for SH3-domain interaction, such as 3_10_-helices, but these structures were observed by X-ray crystallography of peptide-SH3-domain complexes that were better-behaved than the one studied here [27,36,72]. We consider that a more realistic scenario here is that MBP samples a variety of conformations within the SH3-domain pocket, and that these are modulated by post-translational modifications, such as phosphorylation by MAPKs at T92 and T95 [8,73]. Although it was expected here that the proline-rich segment of this IDP would form a PPII conformation [41], there are exceptions to this model [74].

We conclude that the interaction of 18.5 kDa MBP with Fyn–SH3 is another example of ‘fuzzy’ complex formation not necessarily involving coupled folding and binding. The ever-increasing plethora of publications on IDPs is revealing interactions that fall outside any new paradigm that is formulated. Two recent examples are noteworthy. First is the fuzzy complex formed between prothymosin α (an IDP) and the kelch domain of Keap1 (Kelch-like ECH associated protein 1) [75]. The ProTα remained highly flexible even when bound to Keap1, and residues flanking the primary binding motif regulate the binding affinity. Second is the non-structural protein 5A of hepatitis C virus interacting with the Bin1-SH3 domain [76]: as here for 18.5 kDa MBP, additional non-canonical binding regions with the propensity to form α-helices are involved, and the α-helicity is diminished in the complex! We have previously noted that 18.5 kDa MBP has local disorder-to-order transitions upon association with phospholipid membranes or actin microfilaments, yet much of the protein remains mobile [21,64,77,78]. The association of MBP with Ca^2+^-calmodulin involves several possible binding targets [39,40,79,80], yet an NMR spectroscopic structural analysis of a simplified MBP–peptide (residues D143–S163, murine 18.6 kDa numbering) demonstrated heterogeneity in the conformations of the calmodulin, and thus in the complexes [81]. It is noteworthy that the C-terminal target on 18.5 kDa MBP did undergo a disorder-to-order (specifically α-helical) transition, though, *in vitro* [39].

### Concluding remarks and biological significance

It has been suggested that MBP peripherally binds to the oligodendrocyte membrane through folding and insertion of its amphipathic α-helices [15,64]. We have shown in molecular dynamic simulation studies that insertion of the central α-helix of MBP potentially presents the adjacent proline-rich region to the cytoplasm for binding to SH3–domains [22,23]. In the context of the discussion by Uversky and Dunker [16], we can argue that, under true physiological conditions, the proline-rich region of MBP does not require a pre-formed PPII structure to bind to Fyn–SH3 *per se*, nor does binding induce its formation. The segment T62–L68 has been found to be mobile and solvent-exposed in a solid-state NMR study of membrane-reconstituted 18.5 kDa MBP [21,77], and thus would also be directly accessible for interaction with Fyn–SH3. While the tertiary conformation of 18.5 kDa MBP within myelin remains unknown, much evidence supports a tertiary fold involving at least a hairpin bend at residue P93 [22,23] which could be instrumental in positioning the T62–L68 segment closer to the proline-rich region, allowing it to participate in binding. The interaction of MBP and Fyn, which would tether the latter to the oligodendrocyte membrane [9], could be modulated by post-translational modifications in the proline-rich region of MBP, especially phosphorylation at T92 and T95. Previous ITC experiments have shown that pseudo-phosphorylation reduces the magnitude of the heats of interaction of full-length 18.5 kDa MBP and Fyn–SH3, suggesting reduced affinity [10]. Molecular dynamic simulations on the MBP-peptide S72–S107 have shown that phosphorylation of residues T92 and/or T95 could potentially link co-operatively the interaction of MBP and Fyn–SH3 in two ways [23]. Firstly, phosphorylation alters the disposition of the central membrane-anchoring α-helix with respect to the membrane which could modify how the proline-rich region is presented to the cytoplasm for binding; and secondly, the formation of salt-bridges involving the phosphorylated threonines and basic residues within the proline-rich region could reduce its flexibility [23]. In light of our finding here that MBP and Fyn–SH3 forms a dynamic fuzzy complex, a reduction in conformational freedom in the SH3–ligand could lead to an altered interaction, beyond affecting simple local electrostatic interactions. In the future, it will be particularly challenging, but necessary, to probe spectroscopically how membrane-associated 18.5 kDa MBP interacts with SH3–domain containing proteins and how combinatorial phosphorylation events can modulate these multifaceted *in situ* interactions. It is important to understand these events further because interference in these normal associations during brain development has been suggested to result potentially in myelin instability and subsequent pathologies [10,82–86].

## Online data

Supplementary data

## References

[1] Bakhti M., Aggarwal S., Simons M. (2013). Myelin architecture: zippering membranes tightly together. Cell Mol. Life Sci..

[2] Young K. M., Psachoulia K., Tripathi R. B., Dunn S. J., Cossell L., Attwell D., Tohyama K., Richardson W. D. (2013). Oligodendrocyte dynamics in the healthy adult CNS: evidence for myelin remodeling. Neuron.

[3] Fulton D., Paez P. M., Campagnoni A. T. (2010). The multiple roles of myelin protein genes during the development of the oligodendrocyte. ASN Neuro.

[4] Harauz G., Boggs J. M. (2013). Myelin management by the 18.5 kDa and 21.5 kDa classic myelin basic protein isoforms. J. Neurochem..

[5] Moscarello M. A., Juurlink B. H. J., Devon R. M., Doucette J. R., Nazarali A. J., Schreyer D. J., Verge V. M. K. (1997). Myelin basic protein, the ‘executive’ molecule of the myelin membrane. Cell Biology and Pathology of Myelin: Evolving Biological Concepts and Therapeutic Approaches.

[6] Harauz G., Ishiyama N., Hill C. M. D., Bates I. R., Libich D. S., Farès C. (2004). Myelin basic protein–diverse conformational states of an intrinsically unstructured protein and its roles in myelin assembly and multiple sclerosis. Micron.

[7] Harauz G., Ladizhansky V., Boggs J. M. (2009). Structural polymorphism and multifunctionality of myelin basic protein. Biochemistry.

[8] Polverini E., Rangaraj G., Libich D. S., Boggs J. M., Harauz G. (2008). Binding of the proline-rich segment of myelin basic protein to SH3-domains–Spectroscopic, microarray, and modelling studies of ligand conformation and effects of post-translational modifications. Biochemistry.

[9] Homchaudhuri L., Polverini E., Gao W., Harauz G., Boggs J. M. (2009). Influence of membrane surface charge and post-translational modifications to myelin basic protein on its ability to tether the Fyn-SH3 domain to a membrane *in vitro*. Biochemistry.

[10] Smith G. S. T., De Avila M., Paez P. M., Spreuer V., Wills M. K. B., Jones N., Boggs J. M., Harauz G. (2012). Proline substitutions and threonine pseudophosphorylation of the SH3 ligand of 18.5 kDa myelin basic protein decrease its affinity for the Fyn-SH3 domain and alter process development and protein localization in oligodendrocytes. J. Neurosci. Res..

[11] Ahmed M. A. M., De Avila M., Polverini E., Bessonov K., Bamm V. V., Harauz G. (2012). Solution NMR structure and molecular dynamics simulations of murine 18.5 kDa myelin basic protein segment (S72-S107) in association with dodecylphosphocholine micelles. Biochemistry.

[12] Smith G. S. T., Homchaudhuri L., Boggs J. M., Harauz G. (2012). Classic 18.5- and 21.5 kDa myelin basic protein isoforms associate with cytoskeletal and SH3-domain proteins in the immortalized N19-oligodendroglial cell line stimulated by phorbol ester and IGF-1. Neurochem. Res..

[13] Boggs J. M., Homchaudhuri L., Ranagaraj G., Liu Y., Smith G. S., Harauz G. (2014). Interaction of myelin basic protein with cytoskeletal and signaling proteins in cultured primary oligodendrocytes and N19 oligodendroglial cells. BMC Res. Notes.

[14] Harauz G., Libich D. S. (2009). The classic basic protein of myelin–conserved structural motifs and the dynamic molecular barcode involved in membrane adhesion and protein-protein interactions. Curr. Protein Peptide Sci.

[15] Harauz G., Libich D. S., Polverini E., Vassall K. A., Dunn B.M. (2013). The classic protein of myelin-conserved structural motifs and the dynamic molecular barcode involved in membrane adhesion, protein-protein interactions, and pathogenesis in multiple sclerosis. Advances in Protein and Peptide Science.

[16] Uversky V. N., Dunker A. K. (2013). The case for intrinsically disordered proteins playing contributory roles in molecular recognition without a stable 3D structure, F1000. Biol. Rep..

[17] Habchi J., Tompa P., Longhi S., Uversky V. N. (2014). Introducing protein intrinsic disorder. Chem. Rev..

[18] Tompa P., Fuxreiter M. (2008). Fuzzy complexes: polymorphism and structural disorder in protein–protein interactions. Trends Biochem. Sci..

[19] Fuxreiter M., Tompa P. (2012). Fuzzy complexes: a more stochastic view of protein function. Adv. Exp. Med. Biol.

[20] Tompa P., Szasz C, Buday L. (2005). Structural disorder throws new light on moonlighting. Trends Biochem. Sci..

[21] Libich D. S., Ahmed M. A., Zhong L., Bamm V. V., Ladizhansky V., Harauz G. (2010). Fuzzy complexes of myelin basic protein: NMR spectroscopic investigations of a polymorphic organizational linker of the central nervous system. Biochem. Cell Biol. (Special issue on Protein Folding: Principles and Diseases).

[22] Polverini E., Coll E. P., Tieleman D. P., Harauz G. (2011). Conformational choreography of a molecular switch region in myelin basic protein–mMolecular dynamics shows induced folding and secondary structure type conversion upon threonyl phosphorylation in both aqueous and membrane-associated environments. Biochim. Biophys. Acta (Biomembranes).

[23] Vassall K. A., Bessonov K., De Avila M., Polverini E., Harauz G. (2013). The effects of threonine phosphorylation on the stability and dynamics of the central molecular switch region of 18.5 kDa myelin basic protein. PLoS ONE.

[24] Mayer B. J. (2001). SH3 domains: complexity in moderation. J. Cell Sci..

[25] Kami K., Takeya R., Sumimoto H., Kohda D. (2002). Diverse recognition of non-PxxP peptide ligands by the SH3 domains from p67(phox). Grb2 and Pex13p, EMBO J..

[26] Fazi B., Cope M. J., Douangamath A., Ferracuti S., Schirwitz K., Zucconi A., Drubin D. G., Wilmanns M., Cesareni G., Castagnoli L. (2002). Unusual binding properties of the SH3 domain of the yeast actin-binding protein Abp1: structural and functional analysis. J. Biol. Chem..

[27] Liu Q., Berry D., Nash P., Pawson T., McGlade C. J., Li S. S. (2003). Structural basis for specific binding of the Gads SH3 domain to an RxxK motif-containing SLP-76 peptide: a novel mode of peptide recognition. Mol. Cell.

[28] Jia C. Y., Nie J., Wu C., Li C., Li S. S. (2005). Novel Src homology 3 domain-binding motifs identified from proteomic screen of a pro-rich region. Mol. Cell. Proteomics.

[29] Li S. S. (2005). Specificity and versatility of SH3 and other proline-recognition domains: structural basis and implications for cellular signal transduction. Biochem. J..

[30] Rath A., Davidson A. R., Deber C. M. (2005). The structure of ‘unstructured’ regions in peptides and proteins: role of the polyproline II helix in protein folding and recognition. Biopolymers.

[31] Hofmann G., Schweimer K., Kiessling A., Hofinger E., Bauer F., Hoffmann S., Rosch P., Campbell I. D., Werner J. M., Sticht H. (2005). Binding, domain orientation, and dynamics of the Lck SH3-SH2 domain pair and comparison with other Src-family kinases. Biochemistry.

[32] Bauer F., Schweimer K., Meiselbach H., Hoffmann S., Rosch P., Sticht H. (2005). Structural characterization of Lyn-SH3 domain in complex with a herpesviral protein reveals an extended recognition motif that enhances binding affinity. Protein Sci..

[33] Liu J., Li M., Ran X., Fan J. S., Song J. (2006). Structural insight into the binding diversity between the human Nck2 SH3 domains and proline-rich proteins. Biochemistry.

[34] McDonald C. B., Seldeen K. L., Deegan B. J., Farooq A. (2009). SH3 domains of Grb2 adaptor bind to PXpsiPXR motifs within the Sos1 nucleotide exchange factor in a discriminate manner. Biochemistry.

[35] Demers J. P., Mittermaier A. (2009). Binding mechanism of an SH3 domain studied by NMR and ITC. J. Am. Chem. Soc..

[36] Harkiolaki M., Lewitzky M., Gilbert R. J., Jones E. Y., Bourette R. P., Mouchiroud G., Sondermann H., Moarefi I., Feller S. M. (2003). Structural basis for SH3 domain-mediated high-affinity binding between Mona/Gads and SLP-76. EMBO J..

[37] Xue Y., Yuwen T., Zhu F., Skrynnikov N. R. (2014). The role of electrostatic interactions in binding of peptides and intrinsically disordered proteins to their folded targets. 1. NMR and MD characterization of the complex between c-Crk N SH3 domain and peptide Sos. Biochemistry.

[38] Meneses E., Mittermaier A. (2014). Electrostatic interactions in the binding pathway of a transient protein complex studied by NMR and isothermal titration calorimetry. J. Biol. Chem..

[39] Libich D. S., Harauz G. (2008). Backbone dynamics of the 18.5 kDa isoform of myelin basic protein reveals transient alpha-helices and a calmodulin-binding site. Biophys. J..

[40] Bamm V. V., De Avila M., Smith G. S. T., Ahmed M. A., Harauz G. (2011). Structured functional domains of myelin basic protein: Cross talk between actin polymerization and Ca(2+)-dependent calmodulin interaction. Biophys. J..

[41] Krieger J. M., Fusco G., Lewitzky M., Simister P. C., Marchant J., Camilloni C., Feller S. M., De S. A. (2014). Conformational recognition of an intrinsically disordered protein. Biophys. J..

[42] Smith G. S. T., Paez P. M., Spreuer V., Campagnoni C. W., Boggs J. M., Campagnoni A. T., Harauz G. (2011). Classical 18.5-and 21.5 kDa isoforms of myelin basic protein inhibit calcium influx into oligodendroglial cells, in contrast to golli isoforms. J. Neurosci. Res..

[43] Barbarese E., Brumwell C., Kwon S., Cui H., Carson J. H. (1999). RNA on the road to myelin. J. Neurocytol..

[44] Verity A. N., Bredesen D., Vonderscher C., Handley V. W., Campagnoni A. T. (1993). Expression of myelin protein genes and other myelin components in an oligodendrocytic cell line conditionally immortalized with a temperature-sensitive retrovirus. J. Neurochem..

[45] Foster L. M., Landry C., Phan T., Campagnoni A. T. (1995). Conditionally immortalized oligodendrocyte cell lines migrate to different brain regions and elaborate ‘myelin-like’ membranes after transplantation into neonatal *shiverer* mouse brains. Dev. Neurosci..

[46] Smith G. S. T., Chen L., Bamm V. V., Dutcher J. R., Harauz G. (2010). The interaction of zinc with membrane-associated 18.5 kDa myelin basic protein: an attenuated total reflectance-Fourier transform infrared spectroscopic study. Amino Acids.

[47] Maxwell K. L., Davidson A. R. (1998). Mutagenesis of a buried polar interaction in an SH3 domain: sequence conservation provides the best prediction of stability effects. Biochemistry.

[48] Schumann F. H., Riepl H., Maurer T., Gronwald W., Neidig K. P., Kalbitzer H. R. (2007). Combined chemical shift changes and amino acid specific chemical shift mapping of protein–protein interactions. J. Biomol. NMR.

[49] Williamson M. P. (2013). Using chemical shift perturbation to characterise ligand binding. Prog. Nucl. Magn. Reson. Spectrosc..

[50] Kanelis V., Donaldson L., Muhandiram D. R., Rotin D., Forman-Kay J. D., Kay L. E. (2000). Sequential assignment of proline-rich regions in proteins: application to modular binding domain complexes. J. Biomol. NMR.

[51] Delaglio F., Grzesiek S., Vuister G. W., Zhu G., Pfeifer J., Bax A. (1995). NMRPipe: a multidimensional spectral processing system based on UNIX pipes. J. Biomol. NMR.

[52] Keller R. (2007). The Computer-aided Resonance Assignment Tutorial.

[53] Ahmed M. A. M., Bamm V. V., Harauz G., Ladizhansky V. (2007). The BG21 isoform of Golli myelin basic protein is intrinsically disordered with a highly flexible amino-terminal domain. Biochemistry.

[54] Camilloni C., De S. A., Vranken W. F., Vendruscolo M. (2012). Determination of secondary structure populations in disordered states of proteins using nuclear magnetic resonance chemical shifts. Biochemistry.

[55] Kay L. E., Torchia D. A., Bax A. (1989). Backbone dynamics of proteins as studied by 15N inverse detected heteronuclear NMR spectroscopy: application to staphylococcal nuclease. Biochemistry.

[56] Boyd J., Hommel U., Campbell I. D. (1990). Influence of cross-correlation between dipolar and anisotropic chemical shift relaxation mechanisms upon longitudinal relaxation rates of 15N in macromolecules. Chem. Phys. Lett..

[57] Palmer A. G., Fairbrother W. J., Cavanagh J., Wright P. E., Rance M. (1992). Improved resolution in three-dimensional constant-time triple resonance NMR spectroscopy of proteins. J. Biomol. NMR.

[58] De Avila M., Ahmed M. A. M., Smith G. S. T., Boggs J. M., Harauz G. (2011). Modes of SH3-domain interactions of 18.5 kDa myelin basic protein *in vitro* and in oligodendrocytes. Biophys. J..

[59] Bamm V. V., Ahmed M. A., Harauz G. (2010). Interaction of myelin basic protein with actin in the presence of dodecylphosphocholine micelles. Biochemistry.

[60] Libich D. S., Harauz G. (2008). Solution NMR and CD spectroscopy of an intrinsically disordered, peripheral membrane protein: evaluation of aqueous and membrane-mimetic solvent conditions for studying the conformational adaptability of the 18.5 kDa isoform of myelin basic protein (MBP). Eur. Biophys. J..

[61] Granot J. (1983). Determination of dissociation constants of 1-1 complexes from NMR data–Optimization of the experimental setup by statistical analysis of simulated experiments. J. Magn. Reson..

[62] Shojania S., O’Neil J. D. (2006). HIV-1 Tat is a natively unfolded protein: the solution conformation and dynamics of reduced HIV-1 Tat-(1-72) by NMR spectroscopy. J. Biol. Chem..

[63] Bates I. R., Feix J. B., Boggs J. M., Harauz G. (2004). An immunodominant epitope of myelin basic protein is an amphipathic alpha-helix. J. Biol. Chem..

[64] Ahmed M. A. M., Bamm V. V., Harauz G., Ladizhansky V. (2010). Solid-state NMR spectroscopy of membrane-associated myelin basic protein–conformation and dynamics of an immunodominant epitope. Biophys. J..

[65] Yu H., Chen J. K., Feng S., Dalgarno D. C., Brauer A. W., Schreiber S. L. (1994). Structural basis for the binding of proline-rich peptides to SH3 domains. Cell.

[66] Solheim S. A., Petsalaki E., Stokka A. J., Russell R. B., Tasken K., Berge T. (2008). Interactions between the Fyn SH3-domain and adaptor protein Cbp/PAG derived ligands, effects on kinase activity and affinity. FEBS J..

[67] Sigalov A. B., Aivazian D. A., Uversky V. N., Stern L. J. (2006). Lipid-binding activity of intrinsically unstructured cytoplasmic domains of multichain immune recognition receptor signaling subunits. Biochemistry.

[68] Clerici M., Mourao A., Gutsche I., Gehring N. H., Hentze M. W., Kulozik A., Kadlec J., Sattler M., Cusack S. (2009). Unusual bipartite mode of interaction between the nonsense-mediated decay factors. UPF1 and UPF2, EMBO J..

[69] Mittag T., Kay L. E., Forman-Kay J. D. (2010). Protein dynamics and conformational disorder in molecular recognition. J. Mol. Recognit..

[70] Mittag T., Marsh J., Grishaev A., Orlicky S., Lin H., Sicheri F., Tyers M., Forman-Kay J. D. (2010). Structure/function implications in a dynamic complex of the intrinsically disordered Sic1 with the Cdc4 subunit of an SCF ubiquitin ligase. Structure.

[71] Iesmantavicius V., Dogan J., Jemth P., Teilum K., Kjaergaard M. (2014). Helical propensity in an intrinsically disordered protein accelerates ligand binding. Angew. Chem. Int. Ed. Engl..

[72] Harkiolaki M., Tsirka T., Lewitzky M., Simister P. C., Joshi D., Bird L. E., Jones E. Y., O’Reilly N., Feller S. M. (2009). Distinct binding modes of two epitopes in Gab2 that interact with the SH3C domain of Grb2. Structure.

[73] Bui J. M., Gsponer J. (2014). Phosphorylation of an intrinsically disordered segment in Ets1 shifts conformational sampling toward binding-competent substates. Structure.

[74] Makowska J., Rodziewicz-Motowidlo S., Baginska K., Vila J. A., Liwo A., Chmurzynski L., Scheraga H. A. (2006). Polyproline II conformation is one of many local conformational states and is not an overall conformation of unfolded peptides and proteins. Proc. Natl. Acad. Sci. U. S. A..

[75] Khan H., Cino E. A., Brickenden A., Fan J., Yang D., Choy W. Y. (2013). Fuzzy complex formation between the intrinsically disordered prothymosin alpha and the Kelch Domain of Keap1 involved in the oxidative stress response. J. Mol. Biol..

[76] Schwarten M., Solyom Z., Feuerstein S., Aladag A., Hoffmann S., Willbold D., Brutscher B. (2013). Interaction of nonstructural protein 5A of the hepatitis C virus with Src homology 3 domains using noncanonical binding sites. Biochemistry.

[77] Zhong L., Bamm V. V., Ahmed M. A., Harauz G., Ladizhansky V. (2007). Solid-state NMR spectroscopy of 18.5 kDa myelin basic protein reconstituted with lipid vesicles: spectroscopic characterisation and spectral assignments of solvent-exposed protein fragments. Biochim. Biophys. Acta (Biomembranes).

[78] Ahmed M. A. M., Bamm V. V., Shi L., Steiner-Mosonyi M., Dawson J. F., Brown L., Harauz G., Ladizhansky V. (2009). Induced secondary structure and polymorphism in an intrinsically disordered structural linker of the CNS: Solid-state NMR and FTIR spectroscopy of myelin basic protein bound to actin. Biophys. J..

[79] Majava V., Petoukhov M. V., Hayashi N., Pirila P., Svergun D. I., Kursula P. (2008). Interaction between the C-terminal region of human myelin basic protein and calmodulin: analysis of complex formation and solution structure. BMC Struct. Biol..

[80] Majava V., Wang C., Myllykoski M., Kangas S. M., Kang S. U., Hayashi N., Baumgartel P., Heape A. M., Lubec G., Kursula P. (2010). Structural analysis of the complex between calmodulin and full-length myelin basic protein, an intrinsically disordered molecule. Amino Acids.

[81] Nagulapalli M., Parigi G., Yuan J., Gsponer J., Deraos G., Bamm V. V., Harauz G., Matsoukas J., de Planque M. R., Gerothanassis I. P. (2012). Recognition pliability is coupled to structural heterogeneity: a calmodulin intrinsically disordered binding region complex. Structure.

[82] Rubenstein E. (2008). Misincorporation of the proline analog azetidine-2-carboxylic acid in the pathogenesis of multiple sclerosis: a hypothesis. J. Neuropathol. Exp. Neurol..

[83] Tait A. R., Straus S. K. (2008). Phosphorylation of U24 from human Herpes Virus type 6 (HHV-6) and its potential role in mimicking myelin basic protein (MBP) in multiple sclerosis. FEBS Lett..

[84] Bessonov K., Bamm V. V., Harauz G. (2010). Misincorporation of the proline homologue Aze (azetidine-2-carboxylic acid) into recombinant myelin basic protein. Phytochemistry.

[85] Morgan A. A., Rubenstein E. (2013). Proline: the distribution, frequency, positioning, and common functional roles of proline and polyproline sequences in the human proteome. PLoS ONE.

[86] Sang Y., Tait A. R., Scott W. R., Creagh A. L., Kumar P., Haynes C. A., Straus S. K. (2014). Probing the interaction between U24 and the SH3 domain of Fyn tyrosine kinase. Biochemistry.

