# Supplementary material for: The proline-rich region of 18.5 kDa myelin basic protein binds to the SH3-domain of Fyn tyrosine kinase with the aid of an upstream segment to form a dynamic complex in vitro
Source: Biosci Rep. 2014 Dec 8;34(6):e00157. doi: 10.1042/BSR20140149 (PMC4266924; doi:10.1042/BSR20140149)
Supplement: Supplementary data [file bsr034e157ntsadd.pdf]

# SUPPLEMENTARY DATA

## The proline-rich region of 18.5-kDa myelin basic protein binds to the SH3-domain of Fyn tyrosine kinase with the aid of an upstream segment to form a dynamic complex *in vitro*

**Miguel De Avila[1], Kenrick A. Vassall[1], Graham S.T. Smith[1], Vladimir V. Bamm[1], and George Harauz[1,2]**

[1]Department of Molecular and Cellular Biology, University of Guelph, 50 Stone Road East, Guelph, Ontario, N1G 2W1, Canada

[2]To whom correspondence should be addressed: Dr. George Harauz; Phone 519-824-4120 (extension 52535); Email: gharauz@uoguelph.ca

## SUPPLEMENTARY EXPERIMENTS

*Cross-linking of xα2-peptide to Fyn-SH3 domain*−Previously, we have used simple polyacrylamide gel electrophoretic (PAGE) analyses of glutaraldehyde cross-linked MBP-protein complexes to demonstrate the existence and stoichiometry of the interaction [1-3]. Here, the xα2-peptide was incubated with Fyn-SH3 at different molar ratios (0:1, 1:0, 1:1, 1:2, respectively) for one hour, with and without 0.0035% (w/v) glutaraldehyde, before analysis by Tricine-PAGE (**Figure S2**).

*Isothermal Titration Calorimetry*−Experiments were carried out using a MicroCal iTC200 calorimeter (GE Healthcare). The Fyn-SH3 domain and untagged 18.5-kDa MBP (or peptides) were both prepared as discussed previously [4] by dialyzing in 20 mM HEPES, 100 mM NaCl, pH 7.4. The Fyn-SH3 was concentrated post-dialysis between 0.7 and 1.3 mM, and MBP was maintained in the concentration range 45-80 μM. The ITC experiments were carried out at 22°C (3 independent measurements) and also 37°C (2 independent measurements). The Fyn-SH3 was injected into the sample cell containing MBP (or peptides). All the titrations began with a preliminary injection of 0.5 μL, followed by 1.0-2.0-μL injections with 3-min spacing in between. Data were integrated, and plotted as a function of molar ratio using Origin 7.0 (OriginLab Corporation, Northampton, MA, USA). Data were fit to the one-set of sites model in Origin 7.0, after subtraction of the heats of dilution which were estimated from the last 3-4 injections. In fitting, the first 3-4 points were excluded as these were distorted by aggregation (**Figure S4**).

# SUPPLEMENTARY TABLES

**Table S1.** Primers used for the construction of recombinant RFP-MBP-C1 N-terminal deletion expression vectors used in this study. All primers were ordered from the University of Guelph Laboratory Services Division.

| Primer | Sequence (from 5' to 3') |
|---|---|
| **MBPC1-20** | AGAGAGAGAGATCCGGAGACCATGCCAGGCATGGCTTCC |
| **MBPC1-30** | AGAGAGAGAGATCCGGACACAGAGACACGGGCATCCTTG |
| **MBPC1-40** | AGAGAGAGAGATCCGGAGGGCGCTTCTTTAGCGGTGACAG |
| **MBPC1-50** | AGAGAGAGAGATCCGGACCCAAGCGGGGCTCTGGCAAGGACTC |
| **MBPC1-60** | AGAGAGAGAGATCCGGAACGAGAACTACCCATTATGGCTCCC |
| **MBPC1-70** | AGAGAGAGAGATCCGGACAGAAGTCGCAGCACGGCCGGACCC |
| **MBPC1-common reverse** | GGTATGGCTGATTATGATCAG |

**Table S2.** Summary of solution NMR acquisition parameters.

| Spectrum | Transients | Carrier Frequency (ppm) | | | Sweep Width (ppm) | | | No. of Points | | | Quadrature Detection | |
|---|---|---|---|---|---|---|---|---|---|---|---|---|
| | | **$F_1$** | **$F_2$** | **$F_3$** | **$F_1$** | **$F_2$** | **$F_3$** | **$F_1$** | **$F_2$** | **$F_3$** | **$F_1$** | **$F_2$** |
| HACAN | 8 | 120 | 54 | 4.7 | 44 | 32 | 16 | 72 | 88 | 2048 | S-TPPI | E-A-E |
| HCCH | 8 | 40 | 40 | 4.7 | 75 | 75 | 14 | 88 | 72 | 2048 | S-TPPI | S-TPPI |
| HNCACB | 8 | 40 | 118 | 4.7 | 64 | 25 | 14 | 128 | 40 | 2048 | S-TPPI | S-TPPI |
| CBCA(CO)NH | 8 | 40 | 118 | 4.7 | 64 | 25 | 14 | 128 | 40 | 2048 | S-TPPI | S-TPPI |
| HN(CA)CO | 16 | 175 | 118 | 4.7 | 16 | 25 | 14 | 64 | 40 | 2048 | S-TPPI | S-TPPI |
| HNCO | 16 | 175 | 118 | 4.7 | 16 | 25 | 14 | 64 | 40 | 2048 | S-TPPI | S-TPPI |
| $^{15}N$ – HSQC (Titration) | 8 | 115 | 4.7 | - | 30 | 16 | - | 256 | 2048 | - | E-A-E | - |
| $^{15}N$ – HSQC ($R_1$, $R_2$) | | 115 | 4.7 | - | 30 | 16 | - | 256 | 2048 | - | E-A-E | - |
| $^{13}C$ – HSQC | 8 | 95 | 4.7 | - | 100 | 10 | - | 512 | 2048 | - | E-A-E | - |

**([a] S-TPPI = States-TPPI [5], E-A-E = Echo-Anti-Echo [6].)**

**Table S3.** Summary of interaction parameters (with the Fyn-SH3 domain) of the murine MBP xα2-peptide (residues S38-S107), or full-length, untagged 18.5-kDa murine MBP (A1-R168), obtained by solution NMR spectroscopy and ITC. In fitting NMR titration data, the value of n was fixed to unity due to the closeness of experimental n (±0.05 including errors). In a previous ITC experiment using hexa-histidine-tagged 18.5-kDa murine MBP (176 residues with an LE linker), only the enthalpy could be estimated to be -8 kcal/mole at a 1:1 molar ratio, but the dissociation constant could not be thus determined [4]. The $K_d$ and $\Delta H$ values reported for the full-length protein are "apparent" values, estimated from fitting ITC data to a one-set of sites binding model, rather than true thermodynamic values because the ITC data were slightly perturbed by aggregation involving the first few injection points.

| **Recombinant 18.5-kDa MBP variant** | **Technique** | $K_d$ † | **n** | **ΔH** |
|---|---|---|---|---|
| | | | | |
| full-length 18.5-kDa MBP, hexa-histidine-tagged (A1-R168-LE$H_6$) [4] | ITC titration | n.d. | n.d. | ~8 kcal/mol |
| as above, with point substitutions P93G, P96G, T92E, T95E [4] | *ditto* | n.d. | n.d. | ~4 kcal/mol |
| | | | | |
| xα2-peptide (S38-S107) [this work] | NMR titration, shift of T95, fractional saturation | 4.0 ± 2.5 μM | 1 | n.a. |
| xα2-peptide (S38-S107) [this work] | NMR titration, shift of Y65, fractional saturation | 7.6 ± 4.6 μM | 1 | n.a. |
| full-length 18.5-kDa MBP, untagged (A1-R168) [this work] | ITC titration at 22 °C, one set of sites model | 5 ± 2 μM | 0.6±0.1 | -7.1± 0.7 kcal/mol |
| full-length 18.5-kDa MBP, untagged (A1-R168) [this work] | ITC titration at 37 °C, one set of sites model | 5±1 μM | 1.2±0.1 | -3.0±0.4 kcal/mol |

† *Apparent not thermodynamic values for ITC of full-length protein.*

## SUPPLEMENTARY FIGURES

**Figure S1. Amino acid sequence of the murine 18.5-kDa MBP isoform (168 residues).** Our original recombinant murine MBP (rmMBP) construct [7,8] had a C-terminal $LEH_6$ tag which was removed here [9]. Here, we also constructed the new extended xα2-peptide, comprising residues (**S38-S107**, in **red** font). The original recombinant construct referred to as the α2-peptide comprised residues (S72-S107), and is indicated by a red line (________) above the sequence [10,11]. This murine 18.5-kDa MBP sequence is compared explicitly with the human and bovine 18.5-kDa isoforms, along with a compendium of post-translational modifications, elsewhere in recent reviews [12,13]. Here, it is noted that the murine T92 and T95 residues represent MAP-kinase targets (highlighted further as **T**), and we have previously studied the effects of phosphorylation at these residues on folding and membrane interactions [14]. The consensus P-x-x-P SH3-ligand is delineated by the symbols "**<-SH3->**".

**ASQKR**$^{005}$ **PSQRS**$^{010}$ **KYLAT**$^{015}$ **ASTMD**$^{020}$ **HARHG**$^{025}$ **FLPRH**$^{030}$
**RDTGI**$^{035}$ **LDSIG**$^{040}$ **RFFSG**$^{045}$ **DRGAP**$^{050}$ **KRGSG**$^{055}$ **KDSHT**$^{060}$

**RTTHY**$^{065}$ **GSLPQ**$^{070}$ **KSQHG**$^{075}$ **RTQDE**$^{080}$ **NPVVH**$^{085}$ **FFKNI**$^{090}$

**VTPRT**$^{095}$ **PPPSQ**$^{100}$ **GKGRG**$^{105}$ **LSLSR**$^{110}$ **FSWGA**$^{115}$ **EGQKP**$^{120}$
**<-SH3->**
**GFGYG**$^{125}$ **GRASD**$^{130}$ **YKSAH**$^{135}$ **KGFKG**$^{140}$ **AYDAQ**$^{145}$ **GTLSK**$^{150}$
**IFKLG**$^{155}$ **GRDSR**$^{160}$ **SGSPM**$^{165}$ **ARR**$^{168}$

**Figure S2. Polyacrylamide gel electrophoretic assay of cross-linked complexes of xα2(S38-S107) and Fyn-SH3**. Tricine-PAGE of xα2(S38-S107) MBP incubated with Fyn-SH3 at different molar ratios. The proteins at each molar ratio were incubated for one hour at room temperature, in a buffer consisting of 10 mM HEPES-NaOH, pH 7.4, with and without 0.0035% (w/v) glutaraldehyde as a cross-linking agent [1,3]. The cross-linking reaction was stopped by adding 1 M TRIS-HCl, pH 7.4, to a final concentration of 100 mM to quench the unreacted glutaraldehyde, before preparation for Tricine-PAGE. The molecular mass of the 1:1 complex would be ~16.9 kDa (xα2-peptide is 7.6 kDa, Fyn-SH3 domain is 9.3 kDa). Lanes present: 1 – molecular mass marker; 2 – 1:0 xα2-peptide:Fyn-SH3 without glutaraldehyde (*i.e.*, xα2-peptide alone); 3 – 1:0 with glutaraldehyde; 4 – 0:1 (*i.e.*, Fyn-SH3 alone) without glutaraldehyde; 5 – 0:1 with glutaraldehyde; 6 – 1:1 without glutaraldehyde; 7 – 1:1 with glutaraldehyde; 8 – 1:2 without glutaraldehyde; 9 – 1:2 with glutaraldehyde.

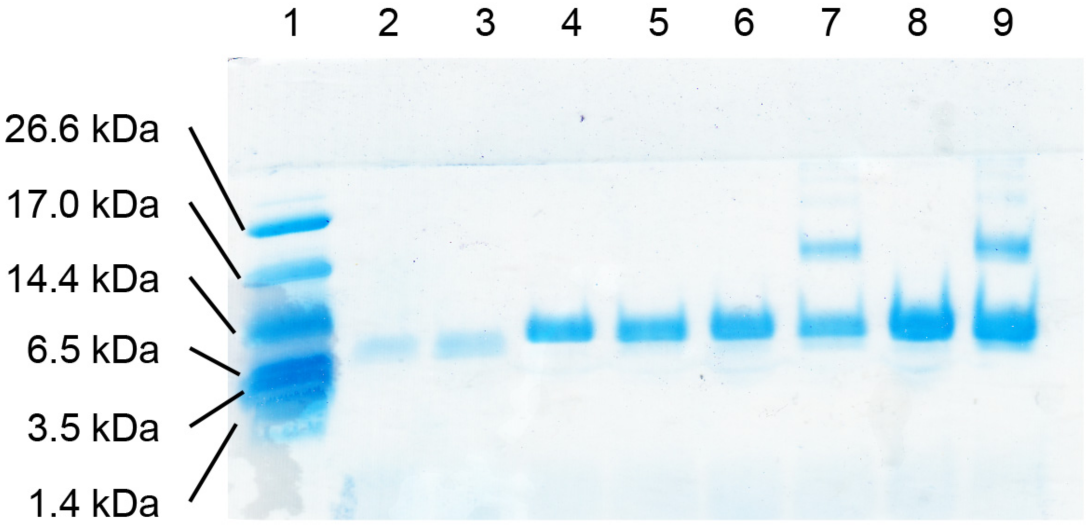

**Figure S3. Chemical shift analysis of secondary structure elements.** Prediction of secondary structure probabilities for each residue (populations per residue) in the xα2-peptide, using the δ2D method designed for disordered proteins [15]. Legend: black (coil), red (poly-proline type II), solid squares (xα2-peptide alone), hollow circles (xα2-peptide in the presence of Fyn-SH3).

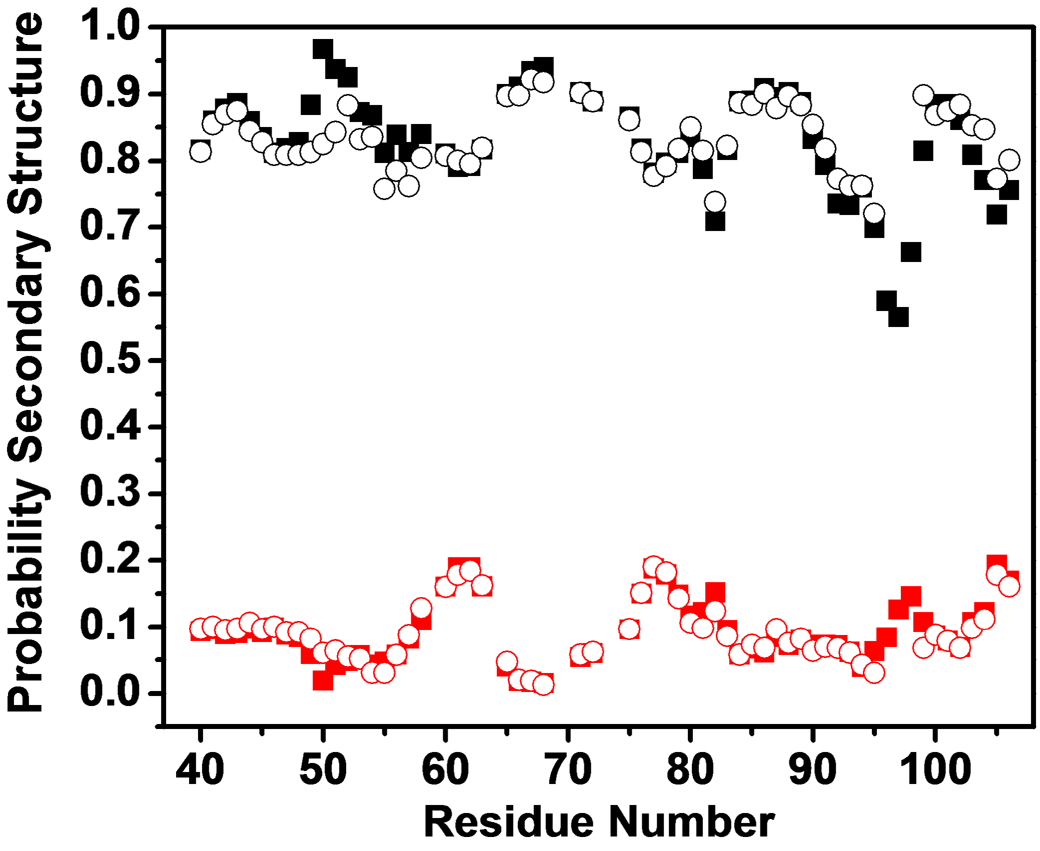

**Figure S4.** Isothermal titration calorimetry (ITC) of full-length murine 18.5-kDa MBP (residues A1-R168) with 9.3-kDa Fyn-SH3 peptide. Representative data of several replicates are shown. Experiments were carried out using a MicroCal iTC200 calorimeter (GE Healthcare). The Fyn-SH3 domain and untagged 18.5-kDa MBP were both prepared as discussed previously [4]. The Fyn-SH3 was concentrated post-dialysis between 0.7 and 1.3 mM, and MBP was maintained in the concentration range 45-80 μM. The ITC experiments were all carried out at (A) 22°C and (B) 37°C. Titrations began with a preliminary injection of 0.5 μL of Fyn-SH3 into the sample cell containing MBP, followed by 1.0-2.0-μL injections with 3-min spacing in between. The top panel shows the heat evolved from each injection of the calorimeter in microcalories/second as a function of time. The bottom panel shows the integration of the area under each peak to plot the heat contribution per mole of Fyn-SH3 as a function of the molar ratio. The ITC data corresponding to the first few injections appear distorted, likely due to the effects of aggregation and were hence excluded from data fitting. The data were fit to a one-set of sites model (Origin 7.0), to obtain **apparent** $K_D$ and $\Delta H$ values (**Table S3**).

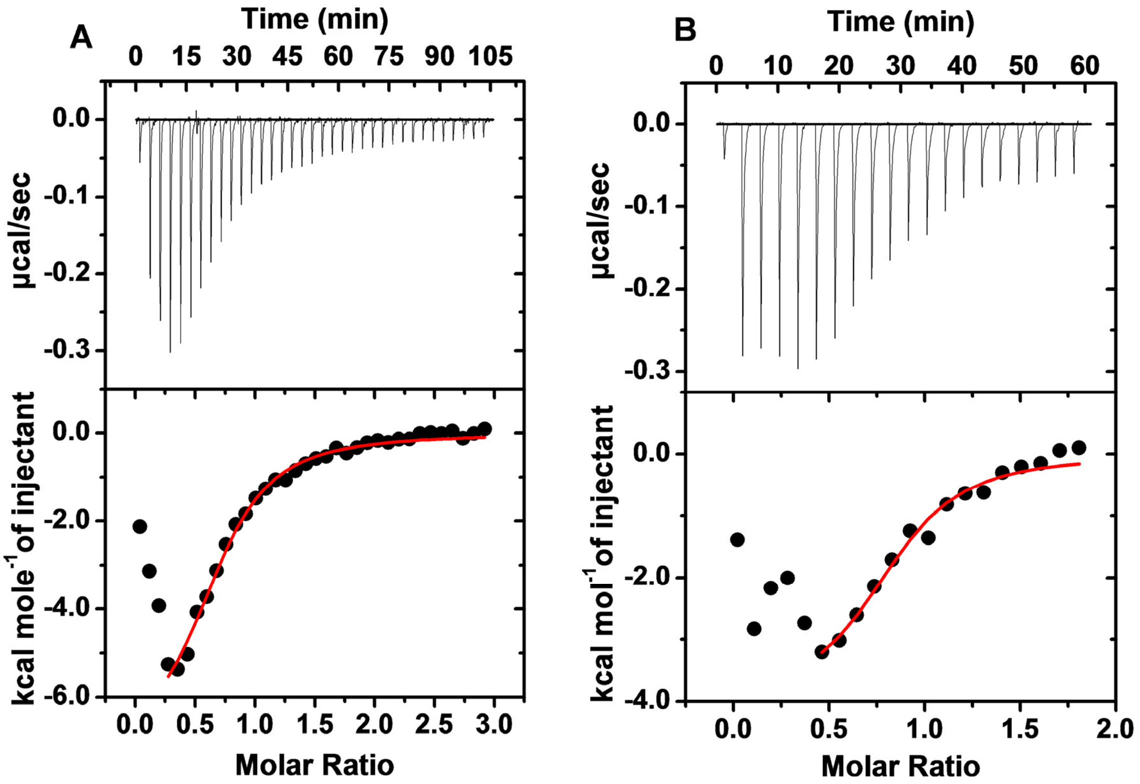

**Figure S5. Chemical shift perturbation of residue T95 of the shorter (S72-S107) α2-peptide upon titration with Fyn-SH3**. Curve showing the weighted chemical shift change of the T95 residue in a shorter peptide of 18.5-kDa MBP containing the proline-rich region. Saturation is not achieved at the same molar ratios observed with the longer (S38-S107) xα2-peptide (*cf.*, **Figure 5** in the main text).

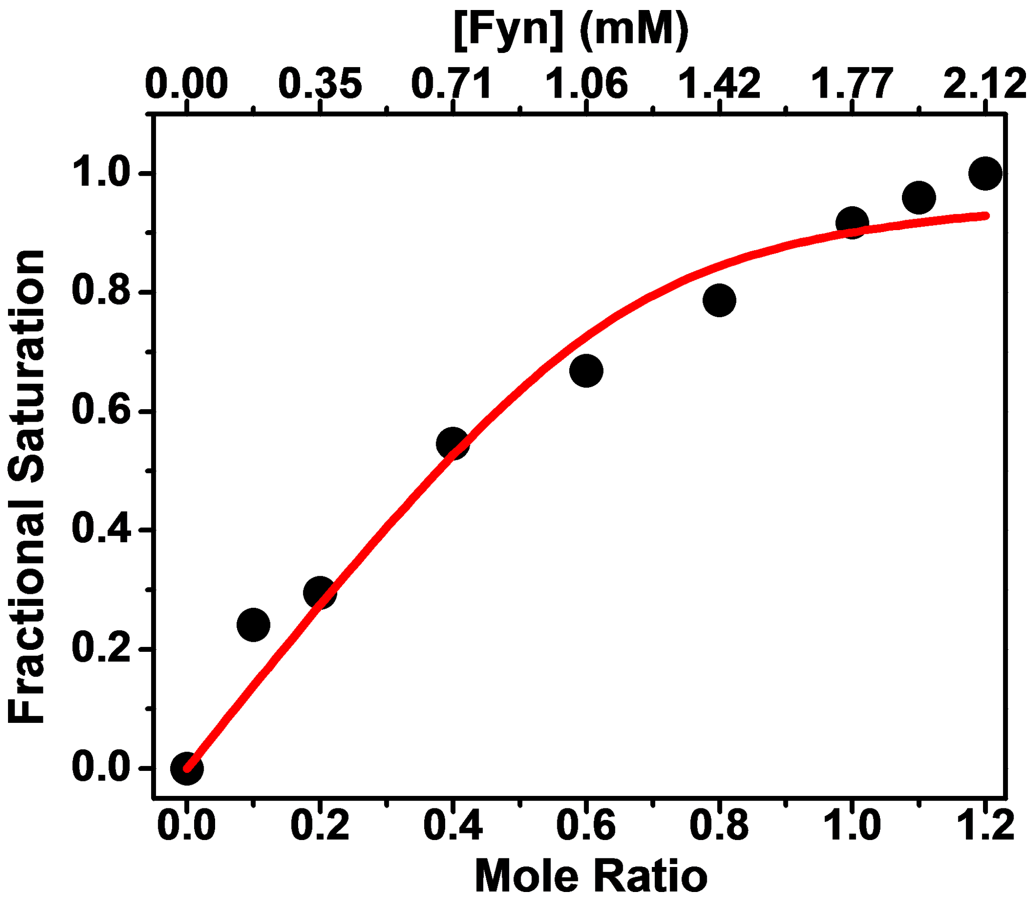

## SUPPLEMENTARY REFERENCES


1 Libich, D.S., Harauz, G. (2002) Interactions of the 18.5-kDa isoform of myelin basic protein with Ca2+-calmodulin - *in vitro* studies using gel shift assays, Mol. Cell. Biochem. **241**, 45-52.

2 Bamm, V.V., Harauz, G. (2008) Expression and purification of the active variant of recombinant murine Golli-interacting protein (GIP) - characterization of its phosphatase activity and interaction with Golli-BG21, Protein Expr. Purif. **62**, 36-43.

3 Bamm, V.V., Ahmed, M.A., Ladizhansky, V., Harauz, G. (2007) Purification and spectroscopic characterization of the recombinant BG21 isoform of murine golli myelin basic protein, J. Neurosci. Res. **85**, 272-284.

4 Smith, G.S.T., De Avila, M., Paez, P.M., Spreuer, V., Wills, M.K.B., Jones, N., Boggs, J.M., Harauz, G. (2012) Proline substitutions and threonine pseudophosphorylation of the SH3 ligand of 18.5-kDa myelin basic protein decrease its affinity for the Fyn-SH3 domain and alter process development and protein localization in oligodendrocytes, J. Neurosci. Res. **90**, 28-47.

5 Marion, D., Ikura, M., Tschudin, R., Bax, A. (1989) Rapid recording of 2D NMR spectra without phase cycling - Application to the study of hydrogen-exchange in proteins, Journal of Magnetic Resonance **85**, 393-399.

6 Kay, L.E., Keifer, P., Saarinen, T. (1992) Pure absorption gradient-enhanced heteronuclear single quantum correlation spectroscopy with improved sensitivity, J. Am. Chem. Soc. **114**, 10663-10665.

7 Bates, I.R., Matharu, P., Ishiyama, N., Rochon, D., Wood, D.D., Polverini, E., Moscarello, M.A., Viner, N.J., Harauz, G. (2000) Characterization of a recombinant murine 18.5-kDa myelin basic protein, Protein Expr. Purif. **20**, 285-299.

8 Bates, I.R., Libich, D.S., Wood, D.D., Moscarello, M.A., Harauz, G. (2002) An Arg/Lys-->Gln mutant of recombinant murine myelin basic protein as a mimic of the deiminated form implicated in multiple sclerosis, Protein Expr. Purif. **25**, 330-341.

9 Smith, G.S.T., Chen, L., Bamm, V.V., Dutcher, J.R., Harauz, G. (2010) The interaction of zinc with membrane-associated 18.5 kDa myelin basic protein: an attenuated total reflectance-Fourier transform infrared spectroscopic study, Amino Acids **39**, 739-750.

10 Bamm, V.V., De Avila, M., Smith, G.S.T., Ahmed, M.A., Harauz, G. (2011) Structured functional domains of myelin basic protein: Cross talk between actin polymerization and Ca(2+)-dependent calmodulin interaction, Biophys. J. **101**, 1248-1256.

11 Ahmed, M.A.M., De Avila, M., Polverini, E., Bessonov, K., Bamm, V.V., Harauz, G. (2012) Solution NMR structure and molecular dynamics simulations of murine 18.5-kDa myelin basic protein segment (S72-S107) in association with dodecylphosphocholine micelles, Biochemistry **51**, 7475-7487.

12 Harauz, G., Boggs, J.M. (2013) Myelin management by the 18.5-kDa and 21.5-kDa classic myelin basic protein isoforms, J. Neurochem. **125**, 334-361.

13 Harauz, G., Libich, D.S., Polverini, E. and Vassall, K.A., The classic protein of myelin - Conserved structural motifs and the dynamic molecular barcode involved in membrane adhesion, protein-protein interactions, and pathogenesis in multiple sclerosis. In: B.M.Dunn (Ed.), Advances in Protein and Peptide Science (e-book; http://benthamscience.com/ebooks/9781608054879/index.htm), Bentham Science Publishers, 2013, pp. 1-53.

14 Vassall, K.A., Bessonov, K., De Avila M., Polverini, E., Harauz, G. (2013) The effects of threonine phosphorylation on the stability and dynamics of the central molecular switch region of 18.5-kDa myelin basic protein, PLoS One **8**, e68175-1-19.

15 Camilloni, C., De, S.A., Vranken, W.F., Vendruscolo, M. (2012) Determination of secondary structure populations in disordered states of proteins using nuclear magnetic resonance chemical shifts, Biochemistry **51**, 2224-2231.
